# Multi‐Sensor Origami Platform: A Customizable System for Obtaining Spatiotemporally Precise Functional Readouts in 3D Models

**DOI:** 10.1002/advs.202305555

**Published:** 2024-04-18

**Authors:** Noam Rahav, Denise Marrero, Adi Soffer, Emma Glickman, Megane Beldjilali‐Labro, Yakey Yaffe, Keshet Tadmor, Yael Leichtmann‐Bardoogo, Uri Ashery, Ben M. Maoz

**Affiliations:** ^1^ School of Neurobiology, Biochemistry and Biophysics The George S. Wise Faculty of Life Sciences Tel Aviv University Tel Aviv 69978 Israel; ^2^ Instituto de Microelectrónica de Barcelona (IMB‐CNM, CSIC) Campus UAB Bellaterra Barcelona 08193 Spain; ^3^ Centro de Investigación Biomédica en Red en Bioingeniería Biomateriales y Nanomedicina Madrid 50018 Spain; ^4^ Department of Biomedical Engineering Tel Aviv University Tel Aviv 69978 Israel; ^5^ Sagol Center for Regenerative Medicine Tel Aviv University Tel Aviv 69978 Israel; ^6^ Sagol School of Neuroscience Tel Aviv University Tel Aviv 69978 Israel; ^7^ The Center for Nanoscience and Nanotechnology Tel Aviv University Tel Aviv 69978 Israel

**Keywords:** 3D MEA, bioprinting, origami, sensor integration

## Abstract

Bioprinting technology offers unprecedented opportunities to construct in vitro tissue models that recapitulate the 3D morphology and functionality of native tissue. Yet, it remains difficult to obtain adequate functional readouts from such models. In particular, it is challenging to position sensors in desired locations within pre‐fabricated 3D bioprinted structures. At the same time, bioprinting tissue directly onto a sensing device is not feasible due to interference with the printer head. As such, a multi‐sensing platform inspired by origami that overcomes these challenges by “folding” around a separately fabricated 3D tissue structure is proposed, allowing for the insertion of electrodes into precise locations, which are custom‐defined using computer‐aided‐design software. The *multi‐sensing origami platform* (MSOP) can be connected to a commercial multi‐electrode array (MEA) system for data‐acquisition and processing. To demonstrate the platform, how integrated 3D MEA electrodes can record neuronal electrical activity in a 3D model of a neurovascular unit is shown. The MSOP also enables a microvascular endothelial network to be cultured separately and integrated with the 3D tissue structure. Accordingly, how impedance‐based sensors in the platform can measure endothelial barrier function is shown. It is further demonstrated the device's versatility by using it to measure neuronal activity in brain organoids.

## Graphical abstract

This proposed multi‐sensing origami platform (MSOP) addresses challenges in obtaining functional readouts from 3D bioprinted tissue models. Inspired by origami, it “folds” around a separately fabricated 3D tissue structure, allowing precise insertion of electrodes. Connected to a multi‐electrode array system, it records neuronal activity, measures endothelial barrier function, and demonstrates versatility with organoids.

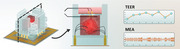

## 1 Introduction

Amid significant advances in the development of in vitro models that closely recapitulate human anatomy and physiology, 3D bioprinting has emerged as a promising approach for engineering such models.^[^ ^1^, ^2^, ^3^ ^]^ With the aid of computer‐aided design (CAD) software, bioprinted models can be produced with high spatial resolution and control, incorporating biocompatible materials, biochemical cues, and heterogeneous cell populations deposited in precise configurations.^[^ ^4^, ^5^ ^]^ Moreover, bioprinting can be used to incorporate vascular networks into tissue, as well as match the biomechanical properties of different organs.^[^ ^6^, ^7^ ^]^ Thus, bioprinting offers a means of producing 3D models comprising complex functional tissue whose similarity to native tissue far exceeds that of traditional in vitro models.^[^ ^8^ ^]^

Still, certain challenges hinder our ability to exploit such models to their fullest potential. One significant challenge is the difficulty in acquiring high‐resolution spatiotemporal functional readouts in situ from 3D bioprinted models—or indeed, from any type of 3D in vitro model (e.g., organoids, assembloids).^[^ ^9^, ^10^ ^]^ Obtaining such readouts is critical for assessing tissue functionality and responses to various toxins, drugs, and chemicals.^[^ ^11^, ^12^, ^13^ ^]^ Established readout systems that are currently used with traditional 2D cell culture architectures are largely ineffective for 3D models: In 2D systems, cells are typically grown directly onto sensors, or on either side of flat membranes, such that sensors can be integrated into device enclosures or beneath the cells. In 3D models, such as cell‐laden hydrogels or organoids, a single layer of 2D electrodes would only have very limited surface contact with the 3D tissue.^[^ ^14^, ^15^, ^16^, ^17^ ^]^ Accordingly, these electrodes might not be able to register signals originating from sites located either too far above or too far below the position in the tissue where the electrodes were placed.

In light of these limitations, researchers have begun to develop designated platforms for real‐time data gathering and label‐free monitoring in advanced 3D cultures.^[^ ^18^ ^]^ Such platforms rely on various approaches. Some include 2D electrodes, which are integrated into the tissue in a layer‐by‐layer manner or are positioned on the device sidewalls. Others include 3D electrodes that extend into the z‐direction or include scaffolds equipped with sensing capabilities.^[^ ^19^, ^20^, ^21^, ^22^, ^23^, ^24^, ^25^, ^26^, ^27^ ^]^ Another approach focuses on the integration of 3D bioprinted models with electronics, giving rise to bioelectronics constructs.^[^ ^28^ ^]^ This strategy relies on the ability to seamlessly print conductive elements alongside biological material, enabling biofeedback and information processing capabilities. Notable examples of this approach include the work of Lind et al., who successfully developed a fully 3D printed device incorporating six functional inks and embedded soft strain gauge sensors.^[^ ^28^ ^]^ Another effort focused on 3D bioprinted constructs containing optical sensor nanoparticles that allowed for the precise 3D mapping of oxygen levels throughout the construct.^[^ ^29^ ^]^ Of late, soft conductive microelectrode arrays tailored for electrophysiological recording of thick cardiac tissues have been described.^[^ ^30^, ^31^ ^]^ The materials used in these systems include the organic semiconductor polymer poly(3,4‐ethylenedioxythiophene) doped with polystyrene sulfonate (PEDOT:PSS), known for its excellent conductivity, biocompatibility, and flexibility. An alternative approach for detection in 3D in vitro systems relies on sensing tools designated for in vivo measurements, such as assessing brain electrophysiology or vasculature impedance^[^ ^32^, ^33^ ^]^.

The systems outlined above, however, come with several shortcomings. In particular, many of these platforms (e.g., microelectrode arrays) involve lengthy and multi‐step cleanroom‐based processes (e.g. lithography, focused ion beam), where design flexibility is restricted and the manufactured device in most cases is not compatible with existing data acquisition hardware and/or software. Moreover, in the case of bioprinted models specifically (as opposed to other types of 3D models), a key challenge arises with regard to the integration of sensing elements into the bioprinting pipeline. On the one hand, if sensors are first embedded into a device onto which tissue is subsequently printed, they may interfere with movement of the printer head. On the other hand, if sensors are embedded after the tissue is printed, it becomes very challenging to position them at a specific desired location.

**Figure 1:**
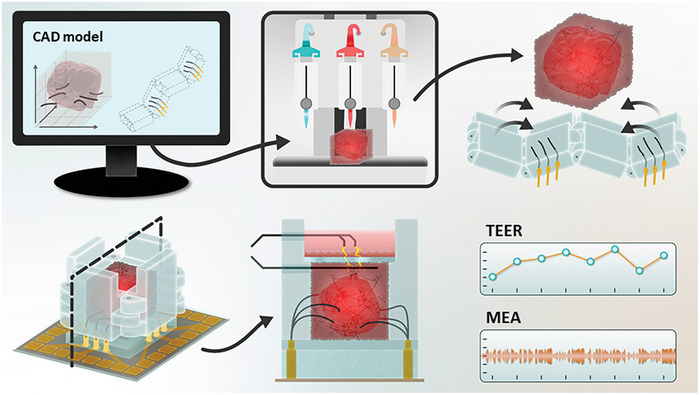
Schematic illustration of the MSOP concept. a) First, the 3D tissue constructs to be modeled and the location of the electrodes within it are designed with CAD software. b) Next, the 3D tissue is bioprinted with the required bioinks. c) Once the tissue is bioprinted, the origami‐inspired PDMS walls are folded around it with the electrodes integrated at pre‐determined locations. d) Next, the folded origami PDMS walls are fitted into an MEA recording system to create the MSOP. e) Cross‐section of the MSOP platform with two types of electrodes mentioned earlier: i) a 3D multielectrode system to record electrophysiological activity at different locations within the 3D model; and ii) impedance‐based electrodes in a tetrapolar configuration for barrier function monitoring. f) Bioelectrical parameters (e.g., TEER and capacitance) and action potential or spikes of electroactive cells from the 3D MEA electrodes are extracted from the MSOP system.

Herein, we put forward a 3D platform that overcomes these hurdles, while vastly expanding the sensing capabilities and application opportunities offered by any individual state‐of‐the‐art approach. Our platform is inspired by origami, the Japanese art of paper folding.^[^ ^34^ ^]^ The basic concept of our approach is to “fold” an electrode‐embedded sensing device around a separately fabricated 3D bioprinted tissue structure, allowing for insertion of the electrodes at precise, custom‐selected coordinates (**Figure** **1**). Moreover, our platform can accommodate multiple types of sensors and incorporate flow. This *multi‐sensor origami platform* (MSOP) is manufactured using standard fabrication techniques and is constructed independently of the bioprinted tissue. As such, it does not interfere with the bioprinting process. The prototype described here incorporates two types of electrodes: i) a 3D multielectrode array (MEA) system with custom positioning to record electrophysiological activity within a 3D tissue sample; and ii) impedance‐based electrodes in a tetrapolar configuration for measuring tissue barrier function.

We validate the platform by constructing and characterizing a model of a neurovascular unit (NVU) comprising a co‐culture of cortical neurons in a 3D configuration in combination with human brain endothelial cells, corresponding to the blood–brain barrier (BBB). Notably, the design of the platform enables the BBB network to be cultured separately from the neurons, and then to be integrated with the 3D neuronal model at any time. This functionality addresses a key challenge facing in vitro modeling of human brain tissues, namely, that microvascular endothelial cells mature at a much faster rate than neuronal cells do, making it difficult to establish co‐cultures in which both cell types are grown simultaneously.^[^ ^13^, ^35^ ^]^ We also demonstrate how the MSOP enables us to monitor the formation and barrier function of the BBB, as well as to assess the electrical functionality of neurons composing the NVU. We further show the capacity of the platform to allow for measurements from distinct locations within a bioprinted 3D structure. Finally, to provide further evidence of platform versatility, we also demonstrate its use with organoids.

The results obtained demonstrate how our system, which can be employed with any cell type or sensor, provides an innovative, yet straightforward means for obtaining spatially resolved functional readouts from 3D bioprinted in vitro models. Though the device described herein is a prototype—which can be further refined and tailored to specific research needs—our findings confirm the MSOP's vast potential in expanding researchers’ access to functional data in vitro.

## 2 Results

### 2.1 Platform Concept and Design Principles

Figure 1 shows a schematic illustration of our 3D, origami‐inspired multi‐sensing platform in combination with a 3D bioprinted tissue structure (the “3D model”). As will be elaborated below, the main component of the MSOP is a set of four connected PDMS walls containing 3D electrodes embedded at specific positions. These so‐called “origami walls” fold around the 3D model, inserting the electrodes into desired locations in the tissue structure. This design enables the 3D model to be fabricated separately from the MSOP, ensuring that platform components do not interfere with the bioprinting process.

As illustrated in the figure, CAD software is used to design the 3D model and to identify the desired locations of the electrodes within it. Next, CAD is used to identify and set the corresponding locations of the electrodes on the origami walls. The components of the MSOP are then assembled (see subsequent section), and the electrodes are positioned in accordance with the CAD design—with the aid of a custom 3D‐printed template derived from the CAD model. The MSOP can then be folded around the 3D model, and measurement can commence. In addition to the 3D electrodes, other types of sensors can be integrated into the platform. Figure 1 shows the configuration we constructed in this report, which includes additional impedance‐based electrodes (see below for further details).

### 2.2 MSOP Components and Assembly

**Figure 2:**
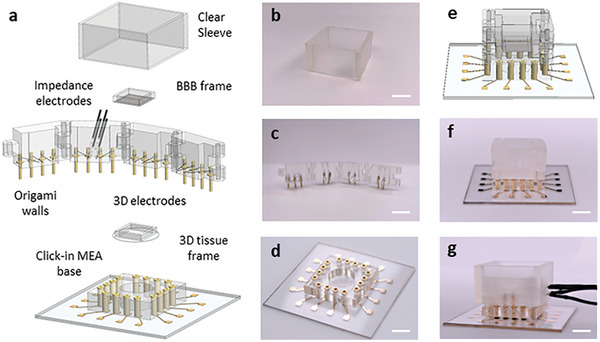
Components of the MSOP system. a) Exploded view in SolidWorks showing all parts of the MSOP with two types of integrated electrodes, namely, 3D MEA electrodes for recording electrical activity in electroactive cells in a 3D model and impedance electrodes to monitor barrier function in a BBB unit integrated within the 3D model. b) Photograph of the clear sleeve used to encase the setup so as to prevent medium leakage (scale bar = 7 mm). c) Photograph of the disassembled origami walls with integrated 3D electrodes (2 electrodes on each wall, scale bar = 10 mm). d) Photograph of the click‐in MEA base compatible with the commercial MEA2100‐Mini head‐stage (scale bar = 8 mm). e) Illustration of the assembled device in SolidWorks (scale bar = 7 mm). f) Photograph of the origami walls folded and inserted into the click‐in MEA base. g) Photograph of the MSOP ready for use with 3D electrodes and impedance‐based electrodes in a tetrapolar configuration (scale bar = 6 mm).

**Figure 3:**
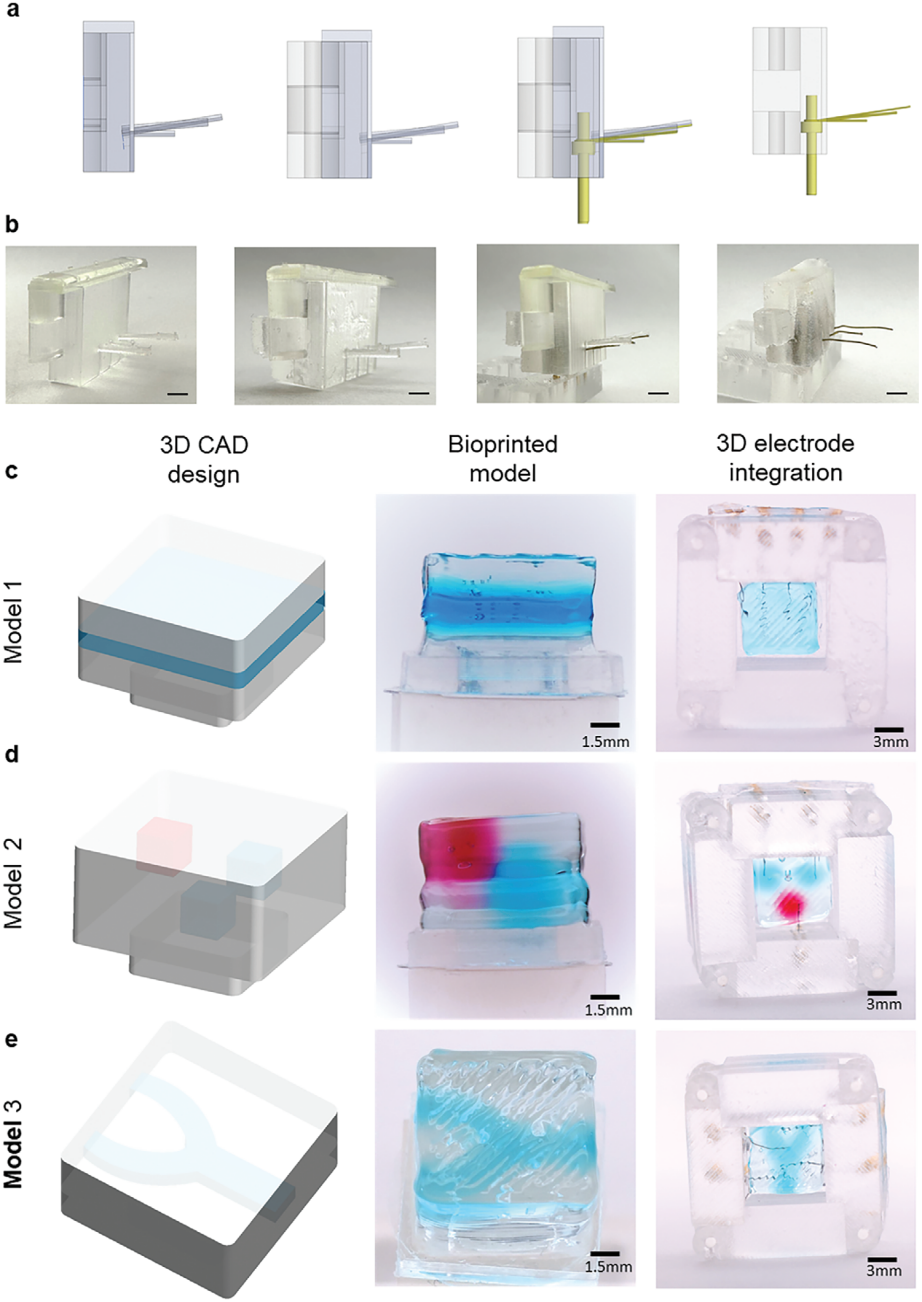
Demonstration of MSOP modularity. Electrode placement can be customized according to the 3D‐printed tissue construct. a) CAD design images of the custom‐designed template, the template inserted into an origami wall, the guided insertion of the electrodes using the template, and an origami wall with embedded electrodes. b) Images corresponding to each step of the CAD model fabrication workflow (scale bar = 6 mm). c) 3D CAD design, bioprinted model and 3D electrode integration of a 3D layer model. d) 3D CAD design, bioprinted model and 3D electrode integration of a three‐organoid model. e) 3D CAD design, bioprinted model and 3D electrode integration of a Y channel model.

**Figure** **2** shows the components of the MSOP and the manner in which they are assembled (see also Figure S9 and Movie S2 Supporting Information; a deconstructed view of the MSOP assembly is shown in Figure 2a). The device includes origami walls (Figure 2c), corresponding to a set of four PDMS walls connected to each other with hinges so as to fold around a 3D model. Each wall contains up to four 3D MEA electrodes (Figure 2a,c; up to 16 electrodes in total), which protrude from the walls at pre‐determined positions. Positioning is achieved as follows: Each electrode is wrapped around a pin, which is then inserted through the bottom of the corresponding wall at the designated position. The electrode is then guided to the desired position along the outside of the origami wall with the aid of an electrode positioning template, custom‐produced (3D‐printed) according to the CAD model (**Figure** **3**a,b; Figures S2 and S3, Supporting Information). In testing the apparatus, we used markers to assess the precision, robustness, and reproducibility of the positioning of the electrodes (Figure S10, Supporting Information); results show that the specific positioning of the electrodes is highly reproducible, with error as low as 220 µm.

**Figure 4:**
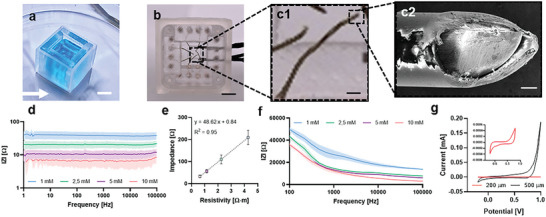
Characterization of 3D and impedance electrodes for integration into the MSOP. a) Demonstration that the MSOP is leak‐tight for long‐term culturing (scale bar = 8.5 mm). b) Photograph of the MSOP showing the integrated impedance and 3D electrodes. Left inset: 3D electrodes. Right inset: Impedance electrodes. c1) Zoom‐in view of a single 3D electrode tip (scale bar = 600 µm). c2) SEM image providing a detailed view of the 3D electrode tip (scale bar = 50 µm). d) Electrochemical characterization of steel electrodes in a tetrapolar configuration for impedance electrodes. e) Cell constant of the tetrapolar impedance electrodes. Impedance was measured with the device assembled and filled with PBS at different resistivities: 4.27, 2.24, 1.15, and 0.64 Ω m, corresponding to a PBS molarity of 1, 2.5, 5, and 10 mM, respectively (*n*  =  3). f) Electrochemical characterization of stainless‐steel electrodes in a two‐point configuration for 3D electrodes. g) Cyclic voltammetry of stainless‐steel electrodes comparing 3D electrodes with 200 µm diameter and impedance electrodes with 500 µm electrode diameter.

The electrodes fit into a click‐in MEA base (Figure 2d), the structure of which matches that of a standard 60‐electrode MEA plate. This design enables the MSOP to be connected to a commercial MEA data acquisition system (MEA2100‐Mini headstage, Multi‐Channel Systems). The base can be used by multiple MSOPs, as it can be plugged in/out for electrical measurement. The 3D model can be mounted, for instance, through bioprinting directly onto a 3D tissue frame, a framed glass coverslip that enables optical inspection under a microscope. This tissue frame is inserted into the center of the base, and the origami walls are then folded around the model and into the base. A clear sleeve (Figure 2b) made of a hard frame material is then placed as a sheath around the assembled device. The sleeve applies even pressure on the origami walls, compressing the origami walls, causing compression and reduction of the inner dimensions in both the x and y directions up to 300 µm each (Figure S11, Supporting Information). This compression ensures a secure seal, thereby preventing medium leakage. In tests in which the assembled MSOP was filled with dyed water and encased in the sleeve (**Figure** **4**a), the assembly remained leak‐tight over the course of the observation period (20 days).

In addition to the 3D MEA electrodes, the MSOP can accommodate different types of sensors for specific applications, such as sensors for measuring impedance, temperature or pH. Herein, we exemplify our platform by constructing a model of the brain NVU. As will be discussed in subsequent sections, our design enabled us to co‐culture neuronal cells in conjunction with a microvascular network, the BBB, which was grown separately on a standalone BBB frame (Figure S12a, Supporting Information). To integrate the BBB into the assembled MSOP, we placed it directly on top of the 3D neuronal sample (Figure S12b, Supporting Information). To measure the vascular barrier function of the BBB, we inserted four wire‐type impedance electrodes through one of the origami walls in a tetrapolar configuration, with two electrodes above and two below the integrated BBB frame. Four small openings were made in the clear sleeve to enable the electrodes to pass through and penetrate the wall.

### 2.3 Customized 3D Sensing for Bioprinted Tissues

A key feature of the MSOP is the capacity to tailor the positioning of 3D MEA electrodes to a specific 3D model. As discussed in previous sections, the precise positioning of the electrodes at specific coordinates is achieved with the aid of an electrode positioning template, which is custom‐produced using CAD software, as illustrated in Figure 3a. Using the CAD software, one can design the template to accommodate any 3D model intended for monitoring, allowing us to specify the number of electrodes and positioning in the 3D structure. The prototypes presented in Figure 3b and Figure S2 (Supporting Information) demonstrate the workflow for positioning electrodes in a sensing wall. When the walls of the MSOP are “folded” around the 3D model, the electrodes penetrate the tissue at the desired locations (Figure 3c,d; Figures S2e and S9, Supporting Information). The high aspect ratio of the 3D electrodes affords the possibility of them being inserted into the tissue without causing any damage, as depicted in Movies S2 and S3 (Supporting Information).

Figure 3c–e and Figure S2e (Supporting Information) provide four examples of bioprinted models together with customized configurations of the MSOP (see Figure S3, Supporting Information for images of the electrode templates used to integrate the electrodes at the specific positions for Figure 3c–e). The models were produced using hydrogel inks of different colors, representing specific regions of interest with different geometries and architectures. For example, model 1 (Figure S3a, Supporting Information) contains a 500 µm‐thick region, colored blue, at the center of the 3D structure. This region could represent a tissue barrier. In this case, the electrodes are all positioned on a single wall of the MSOP, with two electrodes above the blue region and two below it. This setup could enable measurements of tissue barrier permeability. Model 2 (Figure S3b, Supporting Information) consists of three cubes, one red and two blue. In this case, there are two electrodes on one wall of the MSOP, inserted into each of the two blue cubes, while a third electrode on the opposite wall is inserted into the red cube. In model 3 (Figure S3c, Supporting Information), the blue‐dyed region represents the vasculature, with a large vessel splitting into two smaller ones. This model contains four electrodes (two pairs on two opposing walls), all positioned along the vasculature.

The size and viability of 3D tissues significantly depend on the ability to receive oxygen and nutrients (Fick's second law). To use the MSOP with large 3D biostructures, we developed and integrated a perfusion system alongside the 3D electrodes, which can increase oxygenation and nutrient distribution for larger 3D tissues (Figure S8 and Movie S6, Supporting Information). The perfusion system (either syringe‐ or peristaltic pump‐based (Figure S8d, Supporting Information)) can be connected to channels that can be created in the structure. The channels can be fabricated either by 3D bioprinting, or using various other methods, such as needle extraction (Figure S8e,f, Supporting Information). The setup enables the MSOP platform to support constant flow, which ensures effective oxygen and nutrient distribution needed to sustain viable thick tissues. In addition, we characterized the flow dynamics (specifically, the diffusion rate) of a dye in the channel incorporated in the MSOP (Figure S8e–g, Movies S4 and S5, Supporting Information). We saw that the red dye diffused through the hydrogel, traveling a distance of 3 mm after 30 min perfusion (Figure S8g, Supporting Information), resulting in a diffusion rate of ≈0.059 mm min^−1^. It is important to note that these values are highly dependent on the gels, dyes, and environment used in the setup.

### 2.4 Electrode Characterization

Recall that, in our implementation, the device contains two types of electrodes—impedance electrodes and 3D MEA electrodes—both made of stainless steel (Figure 4b, c1). To characterize the electrodes, we performed the following steps. First, we used SEM to characterize the cross‐section of the stainless‐steel electrode tip (Figure 4, c2). As shown in Figure 4, c2, the electrode presents an insulating layer that coats the metal rod. Moreover, it can be seen that the tip is sharp and has a surface area of ≈0.031 mm^2^. As a next step, we recorded the impedance spectra of both types of electrodes (i.e., TEER and MEA) after filling the device with saline solutions of different molarities. Since we wanted to eliminate electrode polarization impedance, we used a tetra‐polar electrode configuration, which allowed us to only consider the impedance of the electrolyte. Thus, the impedance spectra resemble a flat line with decreasing impedance as electrolyte conductivity increases (Figure 4d). Moreover, we obtained the device cell constant (𝐾), which was linear and proportional to electrolyte resistivity with a value of 48.62 m^−1^ (Figure 4e). In contrast to the tetra‐polar measurements, bipolar measurements (Figure 4f) exhibited two regions. At high frequencies, the impedance was directly dependent on the ion solution conductivity and electrode resistance, whereas at low frequencies, the signal was attenuated in a non‐linear fashion by the double‐layer capacitance (Figure 4f). The double‐layer capacitance was directly related to the active surface area of the electrode. Given the marginal standard deviation observed, we can assume that the electrode surface area remained consistent across the different instances tested. In addition, the active areas of the stainless‐steel electrodes (diameters of 200 (3D electrodes) and 500 µm (impedance electrodes)) were measured by cyclic voltammetry (Figure 4g). The 500 µm electrode was shown to have a much larger active surface area than that of the 200 µm electrode.

### 2.5 Measurement of Electrical Activity in a 3D Neuronal Model

**Figure 5:**
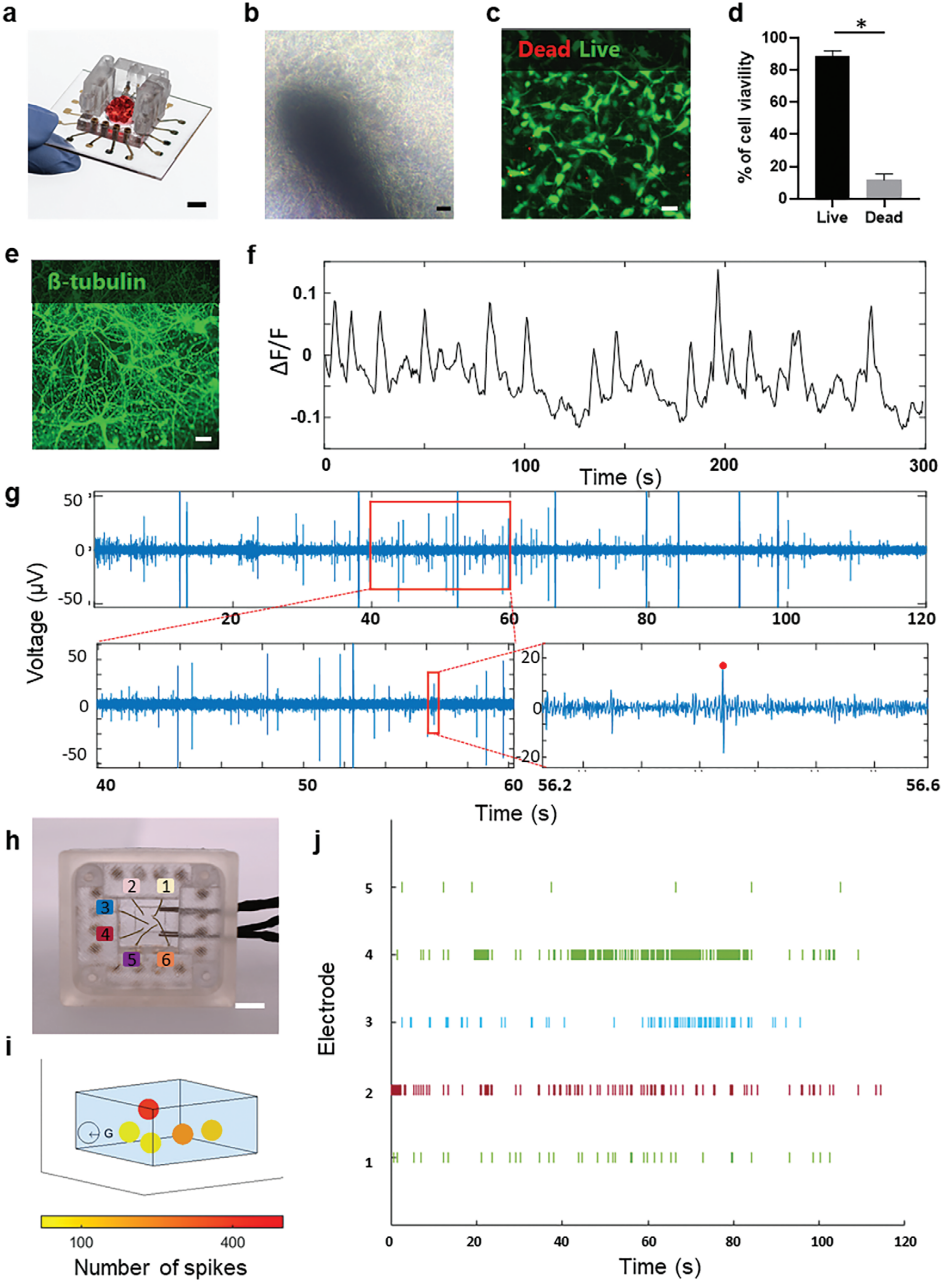
Neuronal modeling in the MSOP system. a) Photograph of the MSOP platform with a bioprinted tissue and integrated 3D electrodes (scale bar = 5.5 mm). b) Microscopy images showing development of neuronal cells on the MSOP after ten days of culturing (scale bar = 50 µm). c) Live/dead images of neurons cultured on the MSOP. d) Cell viability percentage in a long‐term culture of neurons grown in the MSOP (*n*  =  3, scale bar = 30 µm). e) Maximum intensity projections of confocal images of ß‐tubulin immunostaining of neurons cultured in 3D on the MSOP (scale bar = 30 µm) f) Trace of calcium activity of a neuron showing the mean fluorescent intensity of neuronal activity over time. g) Representative spontaneous electrophysiological activity of neurons recorded with the 3D MEA in vitro after 16 days in culture. i) 3D representation of electrical activity per electrode in space (G is the ground electrode) h) Electrode location correspondence with the raster plot electrodes (scale bar = 8.5 mm). j) Raster plot showing spontaneous activity of cultured neurons recorded from the five 3D MEA recording electrodes of the MSOP.

A core function of the MSOP is to facilitate the measurement of electrical activity in electroactive cells in a 3D model. This function is achieved using the 3D MEA electrodes that are positioned on the folding walls of the device and inserted into desired locations in the 3D model. Specifically, these electrodes recorded the local superimposing action potentials of a network of electroactive cells in close proximity to the electrodes. To this end, we prepared the MSOP platform with 3D electrodes able to host 3D bioprinted tissues (**Figure** **5**a). To confirm that the MSOP can indeed allow the measurement of electroactive cells, we first verified that it could host 3D neuronal tissues. The design of the device, in which cells are grown on a glass slide, allows for optical inspection under a microscope and standard assays, such as IHC and calcium imaging (Figure 5). To assess the platform, primary neurons were cultured for up to 20 DIV in MSOPs containing six 3D MEA electrodes. As shown in Figure 5b, a zoomed‐in portion of the 3D culture, highlights the cells’ ability to form a dense network and grow around the 3D electrodes. Live/dead staining assays indicated that primary neuron cultures remained viable in the MSOP, with ≈85% of the cells being alive (Figure 5c,d). IHC confirmed that the cells expressed the neuron‐specific marker of ß‐tubulin and also revealed that the cells were homogeneously spread across the 3D tissue (Figure 5e). These results confirm that the MSOP supports a healthy neuronal culture and that the cells maintain their neuron‐like lineage. Along with morphological and IHC assays, calcium imaging was performed on the 3D tissues to verify that cells were spontaneously electrically active in the MSOP. As shown in Figure 5f, Figures S13–S15, and Movie S6 (Supporting Information), the MSOP allowed us to conduct calcium imaging, which revealed the 3D neuronal cultures to be not only viable but also electrically active.

We next verified the capacity of the MSOP to record electrical activity of 3D cultures. To this end, we used commercially available hardware to record the output of the MSOP‐integrated 3D MEA electrodes in the presence of a neuronal culture. An example of neuronal spiking data for an MSOP containing six integrated 3D MEA electrodes is shown in Figure 5g (electrode number 2 serves as ground (Figure 5h)). All electrodes were active with a heterogeneous firing‐rate distribution, as shown in Figure 5i,j. Figure 5h maps each electrode number to its location in the MSOP, and Figure 5i represents the activity intensity of each electrode in space.

### 2.6 Preparation of the BBB Frame and Assessment of Barrier Function

**Figure 6:**
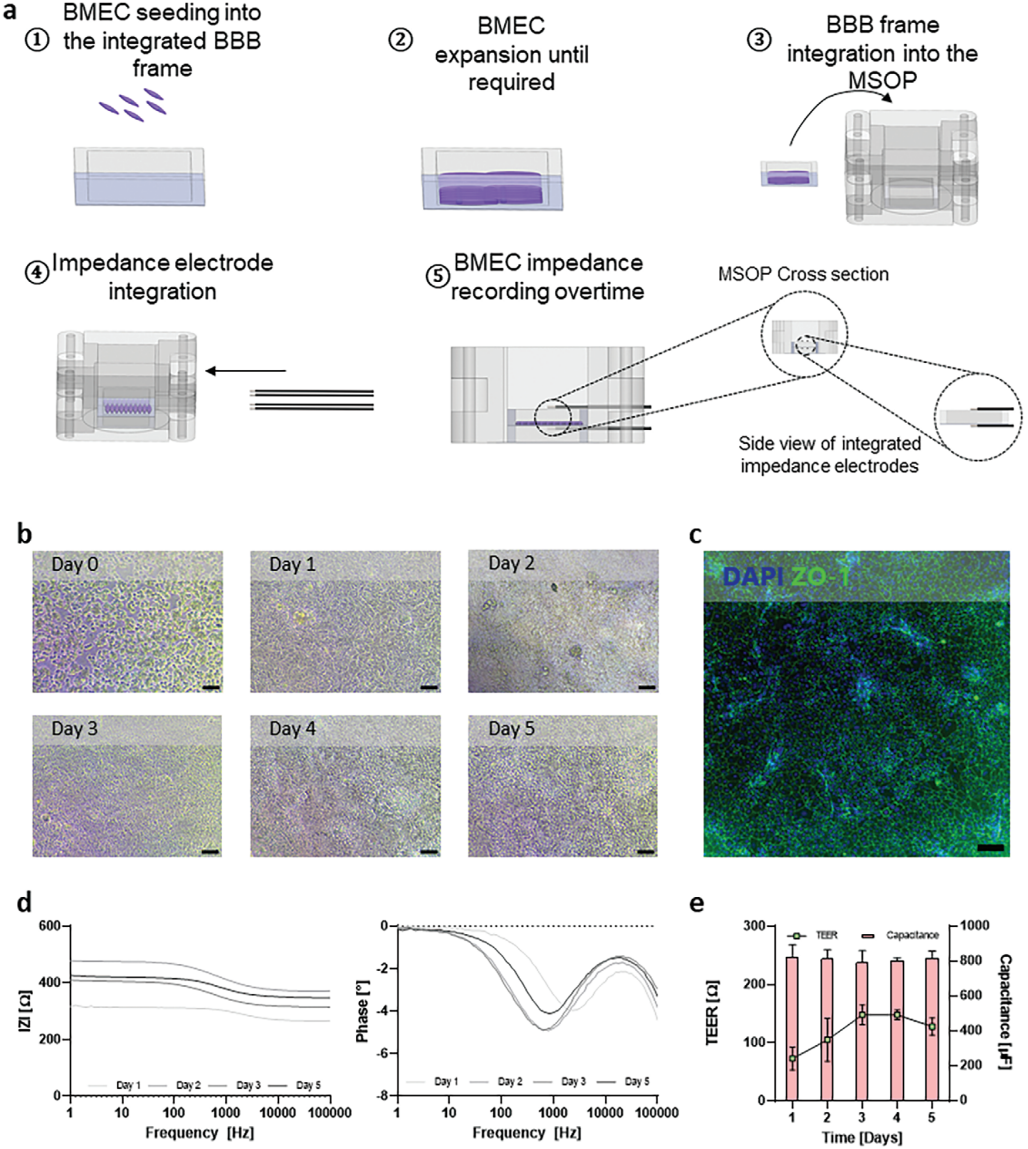
Vascular modeling in the MSOP system. a) Illustration of the vascular component cell culture and barrier function monitoring setup. BMECs are grown on top of the BBB frame to form a confluent monolayer. Then, the BBB frame is introduced inside the MSOP. For barrier function assessment, two electrodes are positioned on either side of the membrane to collect impedance measurements in a tetrapolar configuration. b) Microscopy images showing the development of hiPSC‐derived BMECs from day 1 to day 5 in culture (scale bar = 100 µm). c) Maximum intensity projection of a confocal image of an immunofluorescently labeled vascular monolayer using DAPI (in blue) and anti‐ZO‐1 antibodies (in green) at day 5 in culture (scale bar = 100 µm). d) Experimental impedance data recorded during 5 days of hiPSC‐derived BMEC culture growth inside the MSOP. The vascular barrier function was measured over a frequency range of 1 to 10^5^ Hz. Impedance at low frequencies (<10^2^ Hz) corresponds to the TEER and overall device resistance, while the negative slope at intermediate frequencies (10^2^ – 10^4^ Hz) is related to cellular capacitance. e) TEER and capacitance values of hiPSC‐derived BMECs over time, obtained by fitting the impedance spectra to an equivalent electric circuit.

We designed our platform to enable a microvasculature network, in our case, a BBB, to be prepared as a stand‐alone unit and then integrated into the 3D model when required. The barrier function of the BBB can then be assessed using impedance electrodes arranged in a tetrapolar configuration, with two electrodes above and two below the barrier. In **Figure** **6**a and Figure S12 (Supporting Information), the main steps of the BMEC culturing procedure used to generate the BBB are illustrated. We first prepared the BBB by culturing BMECs in the designated BBB frame. The cells were seeded at high density to enable the rapid formation of a confluent vascular monolayer (Figure 6b). Indeed, it can be seen that the BMECs started to form a tight monolayer with very defined cell‐to‐cell borders at the early stages, due to the increase in transepithelial/transendothelial electrical resistance (TEER). Moreover, immunofluorescence imaging confirmed the presence of ZO‐1 in BMECs forming a tight confluent monolayer (Figure 6c).

We subsequently used impedance measurements (cellular impedance) to evaluate the functionality of the BBB integrated within the MSOP. The recorded spectra of the BMECs displayed three distinct regions (Figure 6d), which are indicators of different physical characteristics of biological barriers. At low frequencies (<100 Hz), the impedance is associated with TEER, which is an indicator of cellular barrier strength. At intermediate frequencies (10^2^–10^4^ Hz), the impedance is related to cellular capacitance. At high frequencies (>10^4^), the contribution is due to the medium and the membrane resistance. As shown in Figure 6e, there was a constant increase in the TEER values, which started at ≈75 Ω after 1 DIV, and reached a peak at day 3, with an average value of 140 Ω. The value remained similar over the next two days. In contrast to TEER, cellular capacitance remained stable and constant at 800 µF for all 5 days. This observation is consistent with the fact that endothelial cells are morphologically flat and do not produce 3D structures on their apical side. Therefore, once the monolayer had been formed on day 1 of culturing, cellular capacitance remained constant for the remainder of the culturing period.

### 2.7 Combined Demonstration: Sensing in a 3D Model of an NVU

**Figure 7:**
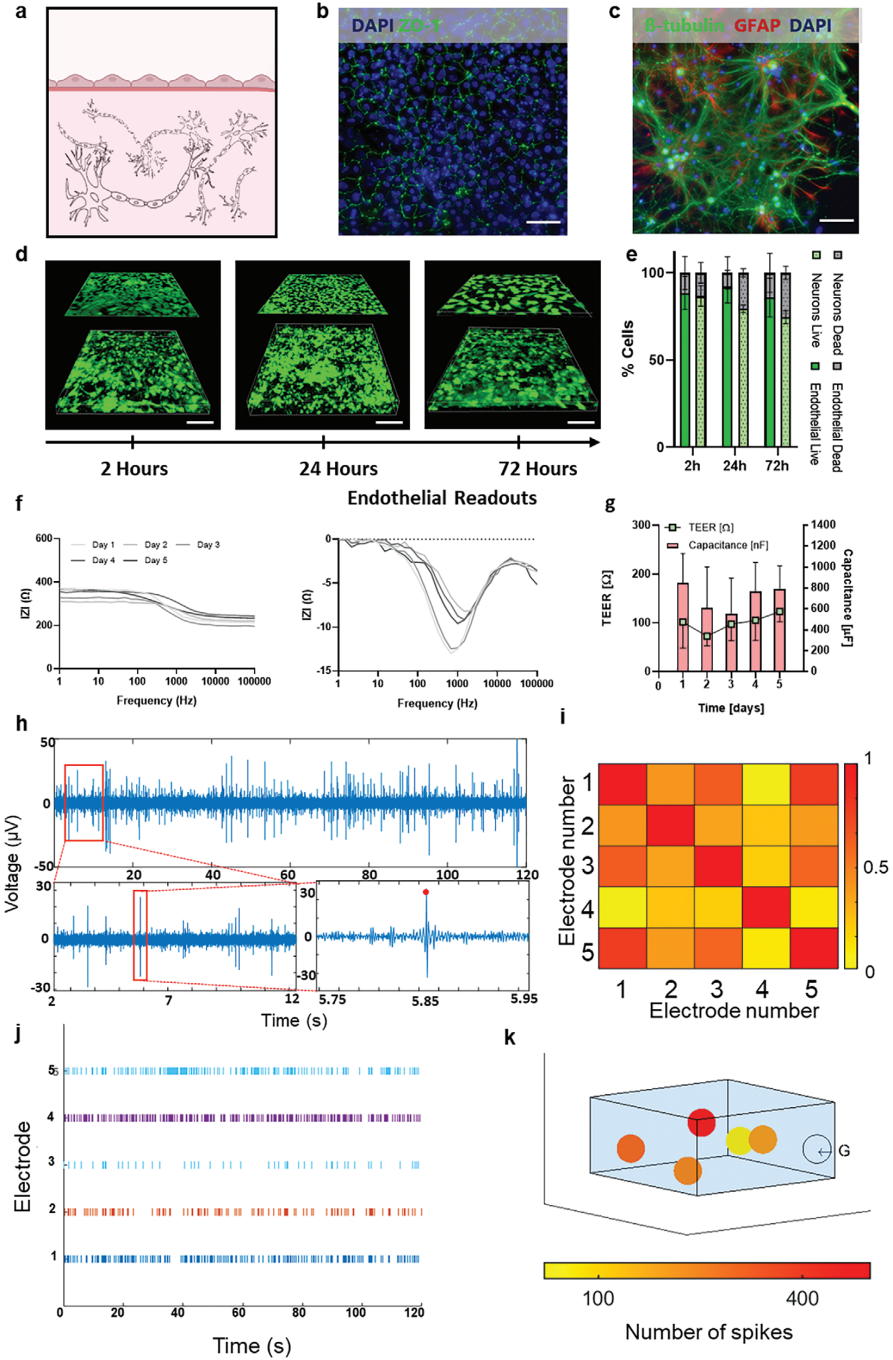
Assessment of an NVU with combined 3D and impedance electrodes in the MSOP. a) Illustration of co‐cultured neurons and BBB cells in the MSOP. b) Maximum intensity projections of confocal images of anti‐Zo‐1 antibody‐ (green) and DAPI‐ (blue) stained BMECs cultured in the MSOP (scale bar = 50 µm). c) Maximum intensity projections of confocal images of anti‐ß‐tubulin (green) and anti‐GFAP antibody‐ (red) and DAPI‐ (blue) stained neurons cultured in 3D in the MSOP (scale bar = 100 µm). d) 3D reconstruction images of the NVU model at different time points. e) Percentage of live and dead endothelial and neuronal cells after 2, 24, and 72 h of co‐culture. f) Experimental impedance data recorded during 5 days of hiPSC‐derived BMECs co‐cultured with neurons in the MSOP. g) TEER and capacitance values of hiPSC‐derived BMECs over time, obtained by fitting the impedance spectra to an equivalent electric circuit. h) Representative spontaneous electrophysiological activity of neurons recorded with the 3D MEA after in vitro 17 days of culture. i) Correlation plot matrix of electrical activity recorded across five electrodes in the MSOP. The numbers 1 to 5 correspond to the electrodes’ indices, while the color scale ranges from 0 to 1, indicating the degree of correlation between electrode pairs. j) Raster plot showing spontaneous activity of cultured neurons recorded from the five 3D MEA recording electrodes of the MSOP. k) 3D mapping of electrical activity per electrode in space (G is the ground electrode).

Having demonstrated that the MSOP can be used to separately measure electrical activity in a 3D neuronal model (using the 3D MEA electrodes embedded in the device walls), and barrier function in the BBB (using impedance electrodes, i.e., TEER and capacitance), we subsequently validated the two functions simultaneously in a comprehensive model of an NVU. The model comprised a 3D culture of neuronal cells in combination with BMECs cultured on the BBB frame (**Figure** **7**a; Figure S12, Supporting Information). Both cell types continued to express their specific markers even when co‐cultured, specifically, ZO‐1 for the BBB and β‐tubulin and GFAP for the 3D neuronal culture (Figure 7b,c). Notably, 80% and 75% of the BBB and the 3D neuronal culture were maintained, respectively, over a three‐day co‐culture period (Figure 7d,e). The NVU model was embedded in the MSOP, and MEA and TEER functionalities were measured in situ. The data obtained correlated with the measurements acquired for the independent compartments (Figures 5 and 6). Specifically, we observed an increase in the BBB's barrier function, as illustrated by increased TEER and capacitance values (Figure 7f,g). We also observed spontaneous electrical activity of the 3D neuronal culture (Figure 7h). Figure 7i shows cross‐correlation between electrodes. It is important to note that the correlation between the electrodes was dependent on their spatial position, where electrode numbers 5 and 1, 3 and 1 were relatively highly correlated. At the same time, electrode numbers 5 and 4, as well as 1 and 4, showed a relatively low correlation. The electrical activity recorded at each electrode is represented as a raster plot in Figure 7j. In addition, a more advanced analysis was performed, which included parameters such as the number of spikes, burst activity, and spike frequency (Figure S16, Supporting Information), as well as mapping of the intensity of neuronal activity at different locations (Figure 7k).

### 2.8 Application of the MSOP with Brain Organoids

**Figure 8:**
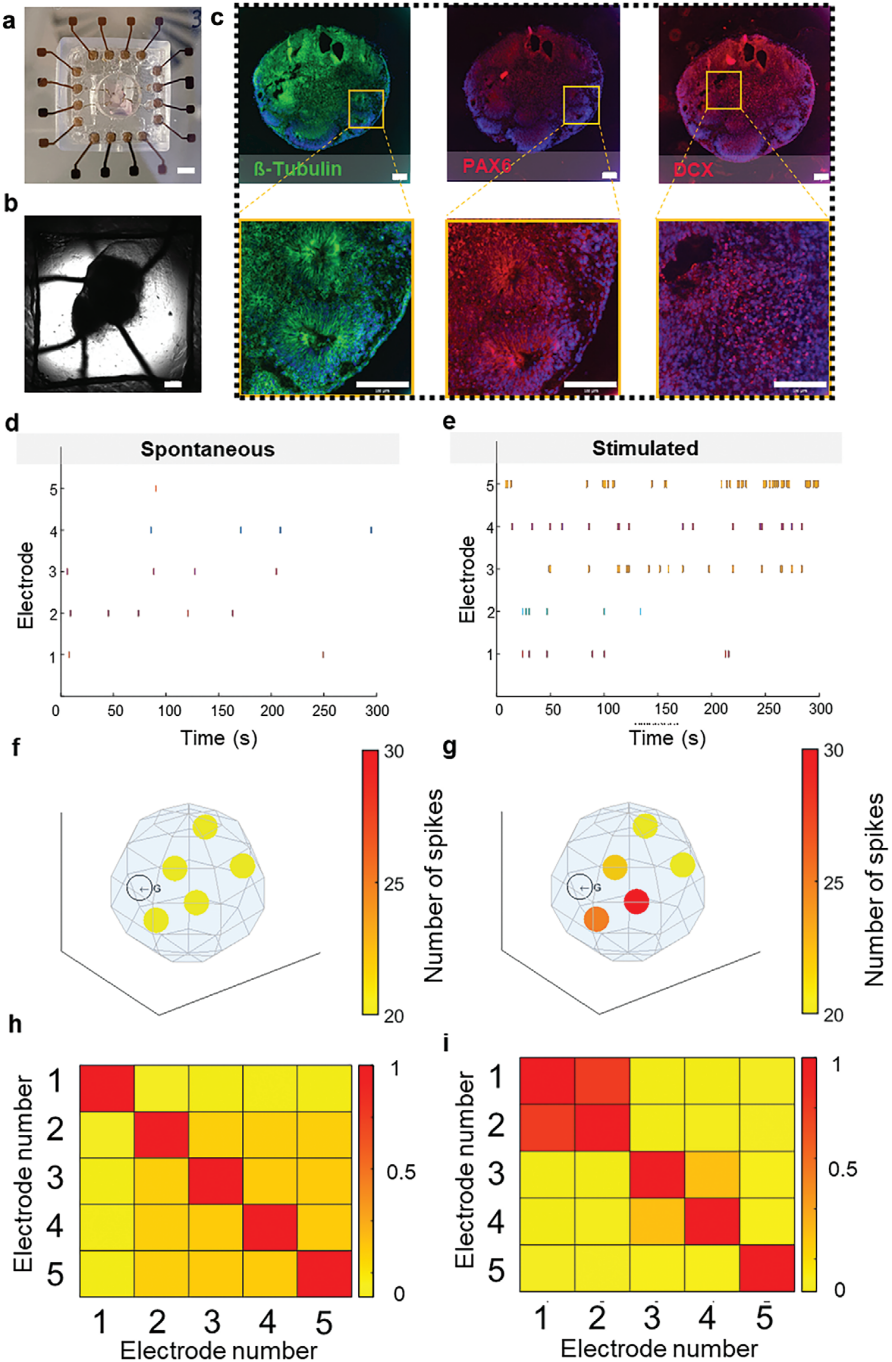
Organoid sensing in the MSOP system. a) Photograph of a brain organoid inside the platform with integrated 3D electrodes for electrophysiology recording (scale bar = 4 mm). b) Top view confocal image of electrodes embedded within a cortical organoid (scale bar = 600 µm). c) IHC for ß‐tubulin (green), PAX6 (red), and DCX (red) in brain organoids (scale bar = 100 µm). d) Raster plot showing spontaneous activity of cultured brain organoids recorded from the six 3D electrodes of the multi‐sensing origami platform. e) Raster plot showing spontaneous activity of cultured brain organoid after being exposed to bicuculline. 3D representations of electrical activity per electrode in space f) before and g) after being exposed to bicuculline. Correlation plot matrices of electrical activity recorded from organoids across five electrodes in the MSOP h) before and i) after exposure to bicuculline.

Clearly, applications of the MSOP are not limited to 3D bioprinted models. The platform can be used to obtain electrophysiological readouts from other types of 3D in vitro models, such as organoids (**Figure** **8**). To demonstrate such an application, we cultured brain organoids in the MSOP for the purpose of recording their neuronal activity (Figure 8a). Movie S6 (Supporting Information) shows a 3D reconstruction of an organoid cultured inside the MSOP. Both Movies S2 and S3 (Supporting Information), as well as Figure 8, show the organoid at the center of the MSOP, with electrodes surrounding the organoid, recording localized electrical activity. IHC (Figure 8c) revealed that organoids cultured in the MSOP expressed the following neuronal markers, indicating successful development and differentiation: i) β‐tubulin, a marker for neuronal differentiation, expression of which suggests that the organoids contain neurons; ii) Pax6, an in vivo marker for the development of the eye and central nervous system, with its presence indicating that the organoids in the MSOP developed as expected; and iii) DCX, a marker for the development of the nervous system, indicative of the organoids containing developing neurons. Readouts from the 3D MEA electrodes (Figure 8d) indicated the presence of action potentials, suggesting that the MSOP successfully recorded spontaneous neuronal electroactivity in the brain organoids. As a further demonstration of the capabilities of the MSOP, we exposed the organoids to bicuculline, a potent GABA_A_ antagonist. We observed that, as expected, electrophysiological activity increased after exposure (Figure 8e). The electrical activity measured at each electrode is shown in Figure 8f,g. Additional electrical activity analysis, including parameters such as the number of spikes, burst activity, and spike frequency, is detailed in Figures S17 and S18 (Supporting Information). Moreover, the correlation between electrodes was calculated (Figure 8h,i). In this context, the increased electrical activity due to the effect of bicuculline is reflected in both the increased number of spikes and by cross‐correlation (Figure 8h,i).

## 3 Discussion

This study proposed and then demonstrated a straightforward solution to an increasingly salient problem, namely, obtaining spatially resolved functional readouts from structurally complex 3D tissue models. Inspired by origami, our multi‐sensing platform folds around a 3D tissue, positioning electrodes at precise coordinates, as can be determined using CAD software in conjunction with the design of the 3D model. Our MSOP also has the ability to incorporate flow to increase the perfusion of oxygen and nutrients for better support of large 3D tissues. We demonstrated the use of the MSOP with a 3D model of an NVU, showing how the platform successfully recorded neuronal electrical activity, in addition to capturing the bioimpedance of vascular barriers. We also showed how the platform can be used with brain organoids cultured directly in the MSOP. Although the current study demonstrated a specific set of applications and MSOP configurations, namely, modeling neuronal and BBB tissue, and obtaining readouts through 3D MEA sensors, as well as impedance sensors, we emphasize that the MSOP can be employed with any type of 3D tissue, as well as with other sensors, such as temperature or pH sensors.

The development of the MSOP required multiple iterations of optimization. A crucial step involved ensuring the precision (i.e., reproducibility) of the electrodes’ positioning, a property that was imperative for the realization of our concept. Multiple approaches were explored before arriving at the final configuration of the electrode positioning template. The templates serve to guide and position the electrodes into specific desired locations designed by the 3D CAD model. Another aspect that required optimization was the clear sleeve, which we discovered was necessary to prevent medium from leaking out of the platform over time. However, the pressure applied by the sleeve led to a displacement of the origami walls by up to 300 µm in both the x and y directions. This offset must be considered when designing templates for electrode integration into the origami walls. The next challenge that had to be met was how to connect the 3D electrodes to a system that would enable their output to be interpreted. To this end, we designed a click‐in MEA base that connects the 3D electrodes to a commercial recording system with an easy, reliable, and repeatable “plug in/plug out” approach. Furthermore, an important feature of the MSOP is the ease of visual access to the in vitro model via a microscope, which enables cellular growth to be monitored, and which facilitates various imaging‐based assays. Finally, it is important to note that since our MSOP can be integrated with perfusion systems, it has the potential to be integrated with vascularized networks in the context of 3D bioprinted architectures.

In this study, we used a tetrapolar electrode configuration to analyze the impedance spectrum of BMECs in the MSOP over time. While most studies provide only TEER values derived from sampling at a single frequency, our system reports, along with TEER, capacitance values by exploiting the full frequency spectrum. With our tetrapolar configuration, we were able to eliminate electrode polarization impedance, which distorts the signal below the cutoff frequency, making data interpretation troublesome and misleading. Eliminating electrode polarization impedance enabled us to ensure that all recorded impedance was directly associated with the monitored tissue. Typically, impedance data are presented as normalized to the total cell culture area. However, we also did so since the generated field across the membrane is not homogeneous (as in most studies), and thus the cell culture area being monitored cannot be accurately determined. Substituting impedance data in Ω cm^2^ for TEER and µF cm^−2^ for cellular capacitance would lead to an overestimation of TEER and an underestimation of capacitance values. For this reason, we presented data in Ω and µF, making data comparison within the same architecture more reliable. It is important to note that impedance measurements are sensitive to changes in temperature, medium conductivity, or electrode placement shifts, and, therefore, it is important to keep all these conditions steady. In our model, the impedance electrodes were located at a single, fixed position. This was achieved by inserting the electrodes through the origami wall, after they first passed through four openings in the clear sleeve. Impedance measurements were carried out in a consistent manner. We minimized recording time and temperature fluctuations by taking the devices out of the incubator for no longer than 2 min and by collecting data from one device at a time.

Regarding neuronal activity, we showed that the readouts acquired from the embedded 3D MEA electrodes captured expected neuronal spiking patterns. The validity of these readouts was supported by the results of calcium imaging assays, a reliable method for verifying cellular electrical activity.^[^ ^42^ ^]^ In future applications, the MSOP offers a preferable alternative to calcium imaging for evaluating electrical activity in 3D models for several reasons. First, calcium imaging provides much lower temporal resolution than does electrode readouts, as calcium fluctuations associated with the depolarization of a cell membrane are much slower than the voltage changes of an action potential. Second, calcium imaging assays have a time limit of approximately two hours, which makes them unsuitable for long‐term monitoring. In contrast, the 3D MEA electrodes of the MSOP can be used over prolonged periods of time, so long as the electrodes remain stable and maintain their electrical properties. Finally, calcium imaging may interfere with biological activity, whereas electrodes are fully biocompatible.^[^ ^43^ ^]^

As noted briefly in the introduction, the fact that our platform is designed to include a stand‐alone BBB frame addresses a major challenge in in vitro modeling of tissues containing multiple cell types, in general, and microvasculature networks, in particular, namely, disparities in cell maturation times. For example, whereas BMECs can mature within a week, neurons must mature for at least 2–3 weeks before they can generate electrical activity. This challenge is further compounded by the fact that, in general, it is difficult to co‐culture BMECs and primary neurons over time, owing to medium incompatibility. Primary neurons are cultured in a specialized medium containing a high concentration of glucose, neuro‐active amino acids, vitamins, and growth factors that support neuronal growth and survival.^[^ ^44^ ^]^ BMECs require a different set of nutrients, including specific endothelial cell growth factors.^[^ ^36^ ^]^ Seeding BMECs on a stand‐alone membrane enables the microvasculature to be integrated with other cell cultures at any desired time, once both cell types are mature. The use of the stand‐alone BBB frame also circumvents uncertainty regarding the quality of BMEC differentiation by allowing for the seeding of BMECs on multiple framed membranes and then selecting the highest‐quality cultures for integration with 3D models.^[^ ^35^, ^45^ ^]^ Our experiments verified that once both cell types had matured, co‐culturing was successful. Specifically, the MSOP was able to simultaneously provide data regarding the impedance and electrical activity of the two cell types, respectively, with our readouts showing that both remained stable and functional.

The usefulness of our platform is not limited to 3D bioprinted tissues. Indeed, we demonstrated the use of the MSOP with organoids, a powerful tool for disease modeling. In such applications, electrodes can be inserted into the organoid at designated locations, and the medium can be changed every day or two. Notably, there are currently only a handful of methods that enable organoid functionality to be monitored in situ over time.^[^ ^46^, ^47^, ^48^ ^]^ While organoids can be extremely sensitive, our demonstration confirmed that the electrodes did not dissociate the organoids. Throughout the duration of the experiment (4 days), the organoids remained viable and functional. Our findings, moreover, confirmed that the electrodes pose no harm to the cultured tissue, demonstrating the compatibility of the MSOP with delicate biological structures.

In summary, we have developed an advanced 3D platform that can be used to embed a wide variety of sensors for obtaining spatiotemporally resolved functional data from 3D models. With this work, we have laid a foundation for customized 3D sensing, in general, and in bioprinted tissue constructs, specifically.

## 4 Experimental Section

### Device Fabrication

All parts of the MSOP were designed using SolidWorks 3D CAD software (SolidWorks, Waltham, MA). The main steps in the MSOP fabrication process are depicted in Figure S1 (Supporting Information). Fabricated components (Figure 2) and their respective functions and assembly are described in detail in the Results section (“MSOP components and assembly”).

To create the clear sleeve (Figure 2a,b), a 4 mm‐thick 28 mm^2^ square was printed with Clear4 resin using a FORM2 resin‐curing 3D printer (Formlabs, Somerville, MA).

To prepare the origami walls (Figure 2a,c), molds were printed with polylactide (PLA) using a Raise 3D Pro2 Dual Extruder 3D Printer (Raise Technologies, Chapel Hill, NC). Molds were filled with polydimethylsiloxane (PDMS) and its curing agent (Sylgard 184, Dow Corning, Midland, MI) at a 10:1 ratio after degassing under vacuum pressure. The parts were cured at room temperature for 24 h. The walls were assembled together with hinges printed with Clear4 resin using a FORM2 resin‐curing 3D printer (Fromlabs).

To generate the click‐in MEA base (Figure 2a,d), polymethyl methacrylate (PMMA) substrates (1 mm‐thick) were cut into a 49 mm^2^ squares using a laser cutting machine (PLS4.75, Universal Laser Systems, Scottsdale, AZ). Electrode patterns were then laser‐cut into the substrate in polyester foil to create a mask for the gold evaporation process. These patterns consisted of 16 tracks, corresponding to the 16 3D electrodes of the array. The tracks serve as an interface between the device electrodes and the MEA recording system. The PMMA substrates and the masks were sonicated with isopropanol (Bio‐Lab, Jerusalem, Israel) and dried with condensed air. The masks were fixed to the PMMA. Next, the substrates were coated with 20 nm of chromium and 200 nm of gold in a sputtering machine. The polyester foil was gently peeled away.

For the standalone BBB frame (Figure 2a), a 2 mm‐thick 8 mm^2^ square PDMS frame was used to support a polycarbonate (PC) membrane (0.4 µm pore size, it4ip, Ottignies‐Louvain‐la‐Neuve, Belgium). Specifically, a thin layer of uncured PDMS was deposited onto the frame and the membrane was placed on top. The frame‐supported membranes were cured for 4 h at 60 °C.

For the 3D tissue frame (Figure 2a), an 8 mm^2^ PDMS frame identical to that used for the BBB frame was affixed to a glass coverslip (13 mm diameter) placed on top, using a thin layer of PDMS, as described for the BBB frame.

For the electrode positioning template (Figure 3a,b; Figures S2 and S3, Supporting Information), templates were designed according to each bioprinted model. The templates were printed with Clear4 resin using a FORM2 resin‐curing 3D printer (Formlabs).

### 3D MEA Sensor Fabrication and Integration

To create the 3D MEA sensors (Figure 2c), which are designed to record electrical activity within a 3D sample, we used 200‐µm diameter coated stainless‐steel wires (304 wire with HML insulation, Surepure Chemetals, Florham Park, NJ), which we cut to the desired lengths. The wires were coiled around gold pins. Then, conductive silver paint (16040, Ted Pella, Redding, CA) cured at room temperature for 10 min was used to bond the wires to the pins. Connections were tested with an amperemeter.

To provide a means of positioning the electrodes in specific desired locations inside a 3D tissue structure, we used a “positioning template” that was 3D printed according to the bioprinted CAD (Figure 3a,b; Figures S2 and S3, Supporting Information). To insert the electrodes into the origami walls, we first created holes with a diameter of 1 mm in the bottom part of each wall (one hole for each electrode) using biopsy punches. Then, the electrode positioning template was fitted into each origami wall. Next, for each electrode, we made a cut along the exterior of the wall to place the electrode in the desired position, guided by the slots of the template. The electrodes were wrapped around gold pins and then inserted through the corresponding wall cuts and fixed with PDMS. Once the electrodes were inserted, the electrode positioning template also ensured that the electrode position was aligned with the biological model.

### Impedance Sensor Fabrication and Integration

To obtain impedance measurements from the standalone BBB frame, we used four coated stainless‐steel wires with HML insulation (500 µm diameter; Surepure Chemetals), which were cut to the desired length. The electrodes were all inserted through the side of a single PDMS origami wall, with two electrodes above the BBB frame and two below it, creating a tetrapolar configuration.

### Electrochemical Sensor Characterization

The electrodes were characterized by electrochemical impedance spectroscopy. Electrode impedance was recorded using a commercially available potentiostat (MTZ‐35, Biologic Science Instruments, Seyssinet‐Pariset, France) driven by EC‐lab software. The impedance was measured using a sinusoidal excitation signal with an amplitude of 10 mV in the 10–100 kHz frequency range. Impedance measurements were carried out using a tetrapolar configuration by connecting the upper left electrode as the working electrode, the lower left electrode as the counter electrode, and the upper right and the lower right electrodes as reference electrodes. To check if the electrodes were responsive to changes in medium conductivity, we recorded the electrode impedance of 10, 5, 2.5, and 1.25 mM phosphate‐buffered saline (PBS). The device cell constant was calculated according to the following expression:

(1) $$K=\frac{R}{\rho }\left[m^{-1}\right]$$

where R is the electrode access resistance in Ω and ρ is the electrolyte resistivity in Ω m.

### Electron Microscopy

A scanning electron microscope (SEM) (GeminiSEM 300 HRSEM, Zeiss, Oberkochen, Germany) was used to study the morphology of stainless‐steel electrodes with HML insulation. The electrodes were secured to a stainless‐steel sample holder using conductive carbon tape. Images of four cut electrode ends were taken under vacuum at 0° and 30° tilt. Voltage was set between 0.400 and 3.00 kV when imaging.

### Endothelial Cell Culture

Brain microvascular endothelial cells (BMECs) were differentiated from human induced pluripotent stem cells (hiPSCs; BGU003i passage 16–18) as described previously, with some modifications.^[^ ^36^, ^37^, ^38^ ^]^ Specifically, hiPSCs were seeded onto Matrigel‐coated plates (Corning, 354234) at 20 800 cells cm^2^ 24 h prior to differentiation. Differentiation was initiated by culturing the cells in DMEM/F12 medium supplemented with 20% knockout serum (Gibco, 10828010), 1% non‐essential An amino acids (Sartorius, 01‐340‐1B), 1 mM L‐glutamine (Gibco), 216 µM β mercaptoethanol (Gibco, 31350‐010), 100 U ml^−1^ penicillin, and 100 µg ml^−1^ streptomycin (Sartorius AG, 03‐031‐1B). Medium was changed daily. After 4 days, the cells were seeded on top of a PC membrane (in the BBB frame; see above) coated with 400 µg ml^−1^ collagen (Sigma–Aldrich, C5533) and 100 µg ml^−1^ fibronectin (Corning, 356008) for 4 h at 37 °C. Cells were cultured in endothelial serum‐free medium (Gibco, 11111044) supplemented with 20 ng ml^−1^ basic fibroblast growth factor (bFGF) (Peprotech, 100–18B), 10 µM retinoic acid (Sigma–Aldrich, R2625), B27 (Gibco, 12587010) and 100 U ml^−1^ penicillin, and 100 µg ml^−1^ streptomycin. After two days of seeding, the medium was replaced with serum‐free medium supplemented with B27 and 100 U ml^−1^ penicillin, and 100 µg ml^−1^ streptomycin. The medium was changed every other day.

### Neuronal Cell Culture

Post‐natal day 1–4 rat pups were sacrificed by decapitation and the cortex was surgically removed in dissection medium (Table S1, Supporting Information). The cortex was chopped into small pieces and enzymatically digested with a buffered digestion medium (Table S2, Supporting Information) containing trypsin (6000 units mL^−1^) and deoxyribonuclease (1560 units mL^−1^) and left at 37 °C, in a 5% CO_2_ atmosphere for 5 min. The first enzyme solution was washed out, and a solution containing a trypsin inhibitor (1000 units mL^−1^) was added for 10 min at 4 °C. The solution was washed out, and a dissection medium containing deoxyribonuclease (1248 units mL^−1^) was added. Next, mechanical dissociation was performed using a 1 mL sterile pipette and then a 200 µL pipette. The released cells were centrifuged at 1300 rpm for 5 min. The supernatant was removed, and the pellet was suspended in plating medium (Table S3, Supporting Information). The cells (700 000) were seeded in the MSOP at a 1‐to‐1 ratio with Matrigel (Corning). After cell seeding, the MSOPs were placed in an incubator for 60 min to allow the cells to accommodate. Then, 350 µL of fresh planting medium was added. The next day, the medium was replaced with neuronal growth medium (Table S4, Supporting Information). The medium was changed daily. After the third day of culturing, the medium was supplemented with 5‐Fluoro‐2′‐deoxyuridine (Fudr) to inhibit glial cell proliferation.

### Derivation and Analysis of Cerebral Organoids

Cerebral organoids were generated as previously described by Y. Elkabetz et al. and D. Rosebrok et al. with slight modifications.^[^ ^39^, ^40^ ^]^ iPSC lines were cultured in Matrigel (100 µg ml^−1^; FAL354234, Corning)‐ coated tissue culture plates (Corning) in Nutristem (05‐100‐1A, Sartorius). For cortical cell differentiation, the cells were grown to 60%–80% confluency and dissociated into a single cell suspension by first incubating with 1 ml EDTA (0.5 mM) for 2 min at 37 °C, followed by replacing the EDTA with 1 ml accutase (per 60 mm culture dish) and incubation for 3 min at 37 °C. The cells were then triturated 10–15 times using p1000 tips until single cells were obtained. The single‐cell suspension was first washed with Nutristem and then with hESC medium, containing DMEM/F12 (01‐170‐1ASartorius), 20% (v/v) knockout serum replacement (01828010; ThermoFisher), 1 mM Glutamax (35050038; Gibco), 100 µM MEM nonessential amino acids (01‐340‐1B; Sartorius), 1% PenStrep (03‐031‐1, B; Sartorius) and 0.1 mM β‐mercaptoethanol (M3148; Sigma–Aldrich) and supplemented with FGF2 (4 ng ml^−1^) and ROCK inhibitor (50 µM; Tocris), after which the cells were centrifuged (270g, 5 min). Single cells were counted and the volume of hESC medium was adjusted, along with FGF2 and ROCK inhibitor, to a concentration of 9000 cells per 150 µl. Suspended single cells were plated onto a 96‐well U‐bottom low‐attachment plate (Corning). The plate was inspected for cell aggregation and formation of embryonic bodies (EBs) on day 1. On day 2, half of the medium was aspirated without disturbing aggregates and 150 µl hESC medium was added to a total of 225 µl hESC medium, along with the appropriate inhibitor molecules SB‐431542 (10 µM), LDN (250 ng ml^−1^) and XAV‐939 (3.3 µM). FGF2 and ROCK inhibitor were withdrawn once the EBs had reached a size of ≈350 µm. On day 4, 150 µl medium was removed and replaced with 150 µl fresh hESC medium, along with the corresponding inhibitor molecules. On day 6, the organoids were transferred into a low‐attachment 24‐well plate along with N2 neural induction medium (DMEM/ F12, 1 mM Glutamax, 100 µM MEM non‐essential amino acids, 1% PenStrep and 1% N2 supplement (17502048; ThermoFisher)). Every second day, 300 µl medium was aspirated and replaced by an equal volume of fresh N2 medium, along with factors, until day 11. On day 11, the organoids (500–600 µm in size) were embedded into 30 µl Matrigel droplets and incubated for 30 min in an incubator, after which they were transferred into a six‐well low‐attachment plate containing N2/NB medium (1:1) along with 1% B27 without RA using a sterile spatula (NB medium containing Neurobasal medium (21103‐049; ThermoFisher), 1 mM Glutamax, 100 µM MEM non‐essential amino acids, 1% PenStrep and 0.1 mM β‐mercaptoethanol). On day 13, the medium was changed with the same medium used on day 11. On day 15, all the medium was removed and replaced with fresh N2/NB medium along with 1% B27 with RA. The organoid dishes were placed on an orbital shaker and the medium was changed daily. For long‐term organoid cultures, the medium was changed every 2 days. Organoids were fixed in 4% paraformaldehyde for 20–40 min at room temperature, and then processed as described under the “Immunohistochemistry” subsection, below.

### Live/Dead Assay

Calcein, AM (C1430, Life Technologies) and ethidium‐bromide (1613016, Bio‐Rad) were used to mark live and dead cells, respectively. Hoechst stain (Hoechst‐33342 H3570, ThermoFisher) was used to label the nuclei of cells. The cells were incubated for 30 min at 37 °C. Imaging was carried out using an inverted confocal microscope (Olympus FV3000‐IX83). Image analysis was done using ImageJ (National Institutes of Health) and Imaris x64 8.4.1 software (Bitplane, South Windsor, CT).

### Ca^+2^ Imaging

Neurons grown in the MSOP were confirmed to display spontaneous electrical activity using intracellular calcium transients. BAPTA‐Oregon green 488 (O6807, Thermo Fisher Scientific) was added to 3D cultured neurons at a concentration of 10 µM resuspended in neuronal medium. First, the medium was removed. Then, the medium containing the fluorescent calcium marker was added and incubated for 40 min at 37 °C. Fluorescence was monitored using an inverted confocal microscope (Olympus FV3000‐IX83). Image analysis was performed using ImageJ software (National Institutes of Health, USA) and Imaris x64 8.4.1 (Bitplane). The region of interest (ROI) for cells was delineated based on the maximum fluorescent intensity observed in the video, and an adjusted threshold was applied using ImageJ. It is noteworthy that the ROI remained consistent throughout the entire video.

### Immunohistochemistry

For Immunohistochemistry (IHC) of endothelial cells, BMECs were stained with fluorophore‐labeled antibodies directed against ZO‐1 (CST‐13663S, Cell Signaling) or VE cadherin (CST‐2500S, Cell Signaling). For 3D neuron cultures, cells were stained with fluorophore‐labeled antibodies directed against beta‐tubulin (ab18207, Abcam) and GFAP (G3893‐100UL, Sigma–Aldrich).

IHC of both types of cells was initiated by transferring the cells to 24‐well plates and washing three times with PBS. The cells were fixed with 4% paraformaldehyde (PFA) in PBS at room temperature (10 min for 2D endothelial cell cultures and 30 min for 3D neuronal cell cultures). After fixation, the cells were washed three more times with PBS and permeabilized with 1% Triton X‐100 in PBS for 20 min. After three washing steps with PBS, the cells were blocked with 1% bovine serum albumin in PBS. The cells were incubated with primary and secondary antibodies in blocking buffer for 1 and 2 h, respectively, with three washing steps in between. After another three PBS washes, nuclei were stained with DAPI (DAPI Fluoromount‐G 0100–20, SoithernBiotech). All steps were performed at room temperature. Imaging was carried out using an inverted confocal microscope (FV3000‐IX83, Olympus) and a fluorescence microscope (BX60MF5, Olympus).

IHC of paraffin‐embedded cortical organoids, was performed as described previously.^[^ ^41^ ^]^ Briefly, day 34 brain cortical organoids were fixed in 4% PFA (Bio‐Lab, Israel), transferred to 4% sucrose solution, dehydrated in increasing concentrations of ethanol and xylene, embedded in paraffin, and sectioned into 5 µm‐thick slices. De‐paraffinized and rehydrated sections were blocked with 5% normal goat serum (Jackson Laboratory, Bar Harbor, ME) in PBS containing 0.1% bovine serum albumin (BSA; Sartorius, Gottingen, Germany). Sections were incubated overnight at room temperature in a humidified chamber with either rabbit anti‐β‐III‐Tubulin (T2200, Sigma–Aldrich, St. Louis, MO), mouse anti‐doublecortin (DCX) (E6, Santa‐Cruz Biotechnology, Dallas, TX) or mouse anti‐PAX6 (Developmental Studies Hybridoma Bank, Iowa City, IA). After washing, Alexa Fluor goat anti‐mouse 594 and goat anti‐rabbit 488 (Thermo Fisher Scientific, Waltham, MA) secondary antibodies were applied for 1 h. Sections were washed with PBS and mounted with DAPI Fluoromount G (SouthernBiotech, Birmingham, AL). Negative controls were incubated with secondary antibodies alone. Image visualization was performed on an Olympus FV3000 confocal microscope with a UPLXAPO 10x objective/0.4NA or UCPLFLN 20x objective/0.7 NA. Images were acquired with the FLUOVIEW software program and analyzed using ImageJ software (National Institutes of Health) and Imaris x64 8.4.1 (Bitplane).

### Impedance Measurements

The impedance of endothelial cells grown on the PC membrane in the BBB frame was recorded using a commercially available potentiostat (MTZ‐35, Biologic Science Instruments,France) driven by EC‐lab software. Impedance was measured using a sinusoidal excitation signal with an amplitude of 10 mV in the frequency range of 1 or 100 Hz to100 kHz. Impedance measurements were carried out using a tetrapolar configuration as described in the subsection of the Results titled “Electrode characterization”. Impedance measurements were performed outside the incubator for a maximum of 1.5 min so as to avoid compromising cell culture.

### Impedance Data Analysis

Impedance measurements obtained from the endothelial cells as described above were fitted to an equivalent electric circuit using the least‐squares method in Python. The equivalent circuit is composed of a constant phase element (CPE) in parallel with a resistor (*R*_TEER_) and in series with another resistor (*R*_s_). Three well‐differentiated regions can be distinguished across the frequency spectrum. At high frequencies (>10^5^ Hz), the impedance is controlled by the cell culture medium conductivity (*R*_s_). At intermediate frequencies (100–10^5^ Hz), impedance is associated with the cell layer capacitance, represented by a CPE. At low frequencies (<100 Hz), impedance is determined by the sum of two ion conductive pathways, namely, the paracellular and transcellular resistances, which is in turn associated with the transepithelial/endothelial electrical resistance (TEER). The mathematical expression for the CPE is:

(2) $$Z_{\mathrm{CPE}}=\frac{1}{k{\left(j\omega \right)}^{\alpha }}$$

where *j* is the imaginary unit, ω is the angular frequency, *k* is the system admittance, and α is an exponent corresponding to 0 or 1 for an ideal resistor or capacitor, respectively. The cellular layer capacitance (*C*_cl_) was estimated using the following equation:

(3) $$\;C_{\mathrm{cl}}=\frac{{\left(K\cdot R_{TEER}\right)}^{\frac{1}{\alpha }}}{R_{TEER}}\;$$

### MEA Measurements

The MEA2100‐system (MEAs, Multi Channel Systems, Reutlingen, Germany), driven by Multi‐Channel Experimenter Software, was used to record electrophysiological activity of neuron cells. Our custom‐made click‐in MEA base (Figure 2a,d) was placed on the MEA2100 headstage, which links the MEA recording system and the 3D sensing device. To preserve cell culture integrity, electrophysiological activity measurements were taken inside the incubator and neuronal cells were recorded in three separate recordings, each spanning 5 min. The recordings were conducted at a sampling rate of 10 kHz and the signal was filtered between 300 and 1100 Hz, the bandwidth where neuronal spike activity, representing voltage changes in the extracellular matrix resulting from neuronal communication through action potentials, occurs. The data were exported as a Hierarchical Data Format 5 (HDF5) file and analyzed using a custom‐made MATLAB (MathWorks, Natick, MA) code.

### Neuronal Activity Analysis

For this study, MEA recording data were obtained and saved in their native format. Subsequently, the data were converted to the HDF5 file format using Multi Channel Data Manager (Ver. 1.14.9.22193) software to ensure compatibility and facilitate further processing. The HDF5 files were loaded into MATLAB for subsequent analysis. To optimize computational efficiency, the data were scaled and down‐sampled by a factor of 4 since the original sampling rate of 10 kHz exceeded the required resolution for single‐well MEA plate analysis.

To extract relevant frequencies for neural activity (spike detection) and eliminate noise originating from power lines and local field potentials (LFP), a finite impulse response (FIR) bandpass filter (BPF) was applied. The FIR BPF was designed as a 3000th‐order filter with a passband of 300 Hz ≤ f ≤ 1100 Hz, which encompasses the typical range of neural activity. Following signal filtration, the objective was to identify the locations of spikes. To achieve this, the “findpeaks” function of MATLAB was utilized, implementing a threshold‐based approach. The threshold was set to 4.5 standard deviations (STD) above and below the average of the entire signal, which was determined through trial and error as being suitable for a specific dataset (Figure S4, Supporting Information). Bursts were defined as ≥ 3 spikes, with ≤ 300 ms inter spike‐interval between spikes on the same burst. To further analyze the data, traces for each electrode were extracted from the processed signals in MATLAB. Additionally, a raster plot of all electrodes was generated to visualize the patterns of neural activity. The electrophysiological analysis process is detailed in the supplementary information (Figure S5, Supporting Information).

Finally, the electrode traces and raster plot were exported as tagged image file (TIF) format files, enabling additional examination and visualization of the processed MEA data. The number of spikes obtained from the analysis was used in the MATLAB code to generate a 3D representation of the electrical activity for 3D culture and the electrodes positions. The costume program can be downloaded from Github (https://github.com/MaozLab/MaozAnalyzer), Supporting file 1, or from https://www.maozlab.com/publications

NeuroExplorer software (Plexon, version 5.429, Texas, USA) was used to compare the results of our analysis conducted using MATLAB with those obtained through the software. For comparison, two BPF were employed: one within the frequency range identical to that specified in our code (300 Hz ≤ f ≤ 1100 Hz), and another covering a higher frequency range (300 Hz ≤ f ≤ 3000 Hz) (Figures S6 and S7, Supporting Information).

### Bioprinting

SolidWorks was used to design 3D structures for bioprinting. The printing properties were set using an open‐source slicer program (Slic3r) from which a g‐code file was generated. A BIO‐X printer (S‐10001‐001, Cellink, Sweden) was used for printing the gel constructs using CELLINK‐Start gel (s20030002, Cellink, Sweden). To produce the printed models, the two inks were sequentially printed within the PDMS 3D tissue frame, supported by a custom‐made platform. Food coloring was used to color the ink to enhance the visibility of the different structures embedded within the printed models. Inks were loaded into 3 ml syringes with a nozzle diameter of 0.410 mm and centrifuged to remove bubbles. Next, the inks were deposited by applying pressure from 30 to 38 mPa and with a printing speed from 1 to 2 mm s^−1^ at room temperature. After each model was printed, the origami walls of a corresponding MSOP (with custom‐designed electrode configuration) were folded around the model, thereby inserting electrodes at the desired positions.

### MSOP with Integrated Flow System

The origami walls were assembled and inserted into the clear sleeve. An 18G sterile needle was inserted through two parallel walls of the system. CELLINK‐Start gel was injected into the open space between the PDMS walls surrounding the needle. The 18G needle was gently removed from the gel and frame, leaving behind an open channel. Two 27G needles attached to 1 mL syringes were then inserted, one on either end of the open channel. One syringe filled with red food coloring was attached to a NE‐4000 Syringe Pump (New Era Pump Systems, Farmingdale, NY), which was used to induce flow at a rate of 0.2 mL min^−1^. Suction was applied by hand using the syringe at the opposite side of the channel to ensure flow through the gel, rather than diffusion.

To further confirm the possibility of utilizing the MSOP in a flow system, the MSOP was attached to a 12‐Channel IP High Precision Multichannel Pump (Cole Parmer, Vernon Hills, IL). TFE teflon tubing (Chemours, Wilmington, DE) with an outer diameter of 1.58 mm, and inner diameter of 0.8 mm was used for the inlet and outlet instead of the 27G needles. This tubing was inserted into PharMed BPT tubing (Saint‐Gobain, Courbevoie, France) with an inner diameter of 2.06 mm. Flow was induced at a rate of 11 µl min^−1^ (Figure S8a–f, Supporting Information).

### Diffusion Analysis in MSOP Channel

The origami walls and clear sleeve were assembled as described above, with an 18G needle attached to a 1 mL syringe filled with red food coloring inserted between two parallel walls. The CELLINK‐Start gel was again injected into the open space between the walls and surrounding the needle. The needle was slowly removed as gentle pressure was applied to the syringe plunger and a small amount of red food coloring was injected into the channel left behind by the 18G needle. Diffusion of the red food coloring was recorded over the next 30 min, and the video was condensed to ≈17 s using iMovie (Apple, Los Altos, CA). Still frames were taken from the recording at 5 min intervals, and the width of the diffusing dye distribution was measured five times/frame at each interval. This process was repeated two more times. The resulting data were plotted and analyzed using GraphPad Prism (GraphPad Software, Boston, MA), including analysis of simple linear regression to determine whether the slopes were significantly non‐zero and significantly different.

### Ethics

All experiments were approved by the local veterinary authority and the animal ethics committee of Tel Aviv University (approval ethic number 01‐19‐079) and performed in accordance with Israeli law. All efforts were made to minimize animal suffering and reduce the number of animals used.

## Conflict of Interest

The authors declare no conflict of interest.

## Supporting information

Supporting Information

Supplemental Movie 1

Supplemental Movie 2

Supplemental Movie 3

Supplemental Movie 4

Supplemental Movie 5

Supplemental Movie 6

## Data Availability

The data that support the findings of this study are available in the supplementary material of this article.
