# Supplementary material for: Multi‐Sensor Origami Platform: A Customizable System for Obtaining Spatiotemporally Precise Functional Readouts in 3D Models
Source: Adv Sci (Weinh). 2024 Apr 18;11(24):2305555. doi: 10.1002/advs.202305555 (PMC11200086; doi:10.1002/advs.202305555)
Supplement: Supplementary file 1 — Supporting Information [file ADVS-11-2305555-s003.pdf]

ADVANCED SCIENCE
Open Access

Supporting Information

for *Adv. Sci.*, DOI 10.1002/advs.202305555

Multi-Sensor Origami Platform: A Customizable System for Obtaining Spatiotemporally Precise Functional Readouts in 3D Models

*Noam Rahav, Denise Marrero, Adi Soffer, Emma Glickman, Megane Beldjilali-Labro, Yakey Yaffe, Keshet Tadmor, Yael Leichtmann-Bardoogo, Uri Ashery and Ben M. Maoz**

Supporting Information for:

Multi-Sensor Origami Platform: A Customizable System for Obtaining Spatiotemporally Precise Functional Readouts in 3D Models

Noam Rahav [†1], Denise Marrero [† 2,3,4], Adi Soffer[4], Emma Glickman[4], Megane Beldjilali-Labro[4], Yakey Yaffe[5], Keshet Tadmor[6], Yael Leichtmann-Bardoogo[4], Uri Ashery[1, 5,6], Ben M. Maoz[*4,5,6,7]

[1] School of Neurobiology, Biochemistry and Biophysics, The George S. Wise Faculty of Life Sciences, Tel Aviv University, Tel Aviv 69978, Israel

[2] Instituto de Microelectrónica de Barcelona (IMB-CNM, CSIC), Campus UAB, Bellaterra 08193, Barcelona, Spain

[3] Centro de Investigación Biomédica en Red en Bioingeniería Biomateriales y Nanomedicina, Madrid, 50018, Spain

[4] Department of Biomedical Engineering, Tel Aviv University, Tel Aviv 69978, Israel

[5] Sagol Center for Regenerative Medicine, Tel Aviv University, Tel Aviv 69978, Israel

[6] Sagol School of Neuroscience, Tel Aviv University, Tel Aviv 69978, Israel

[7] The Center for Nanoscience and Nanotechnology, Tel Aviv University, Tel Aviv 69978, Israel

[†] Authors contributed equally

[*]Correspondence: bmaoz@tauex.tau.ac.il

Figures:

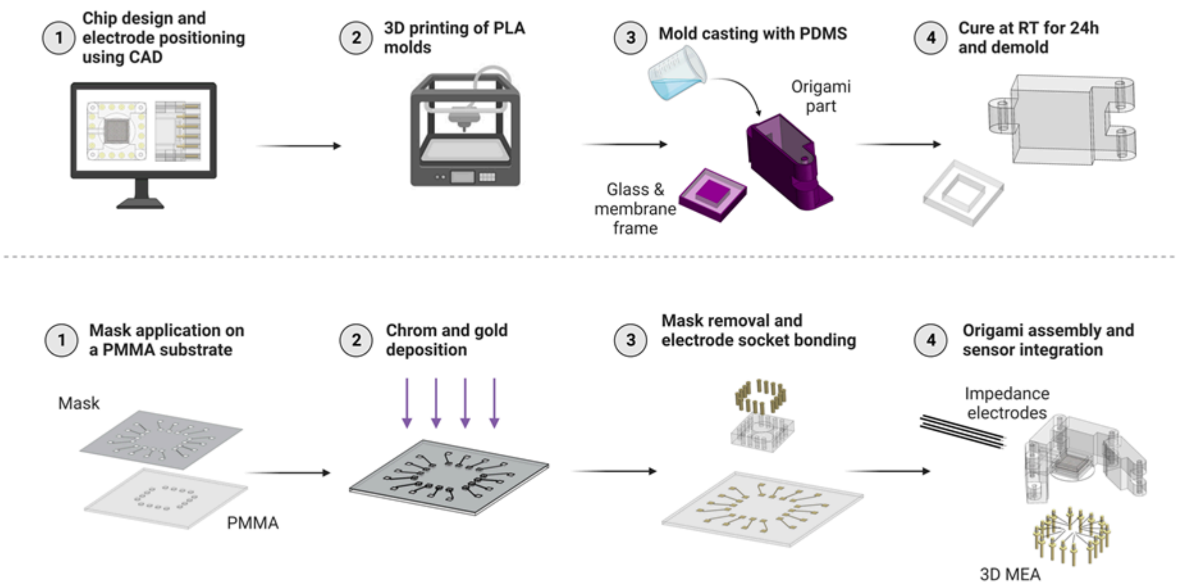

Figure S1. Multi-sensing origami platform fabrication pipeline. Created with Biorender.com

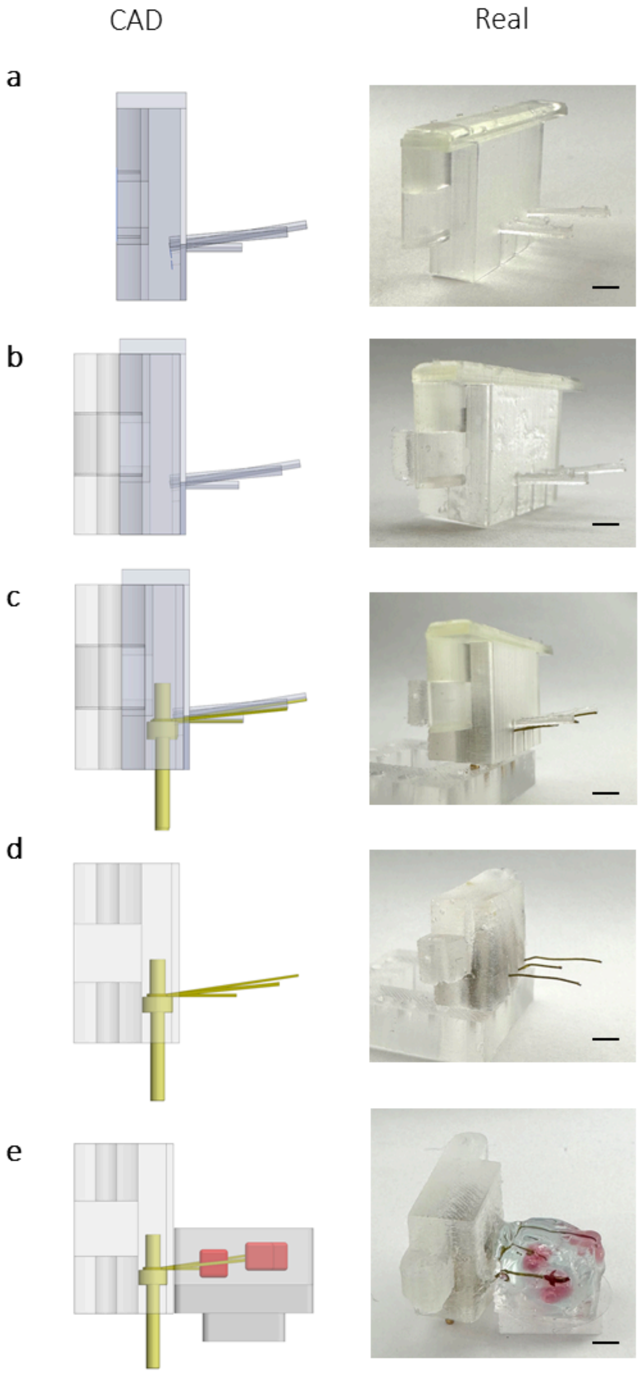

Figure S2. Design of custom template for electrode positioning (scale bar = 6mm).

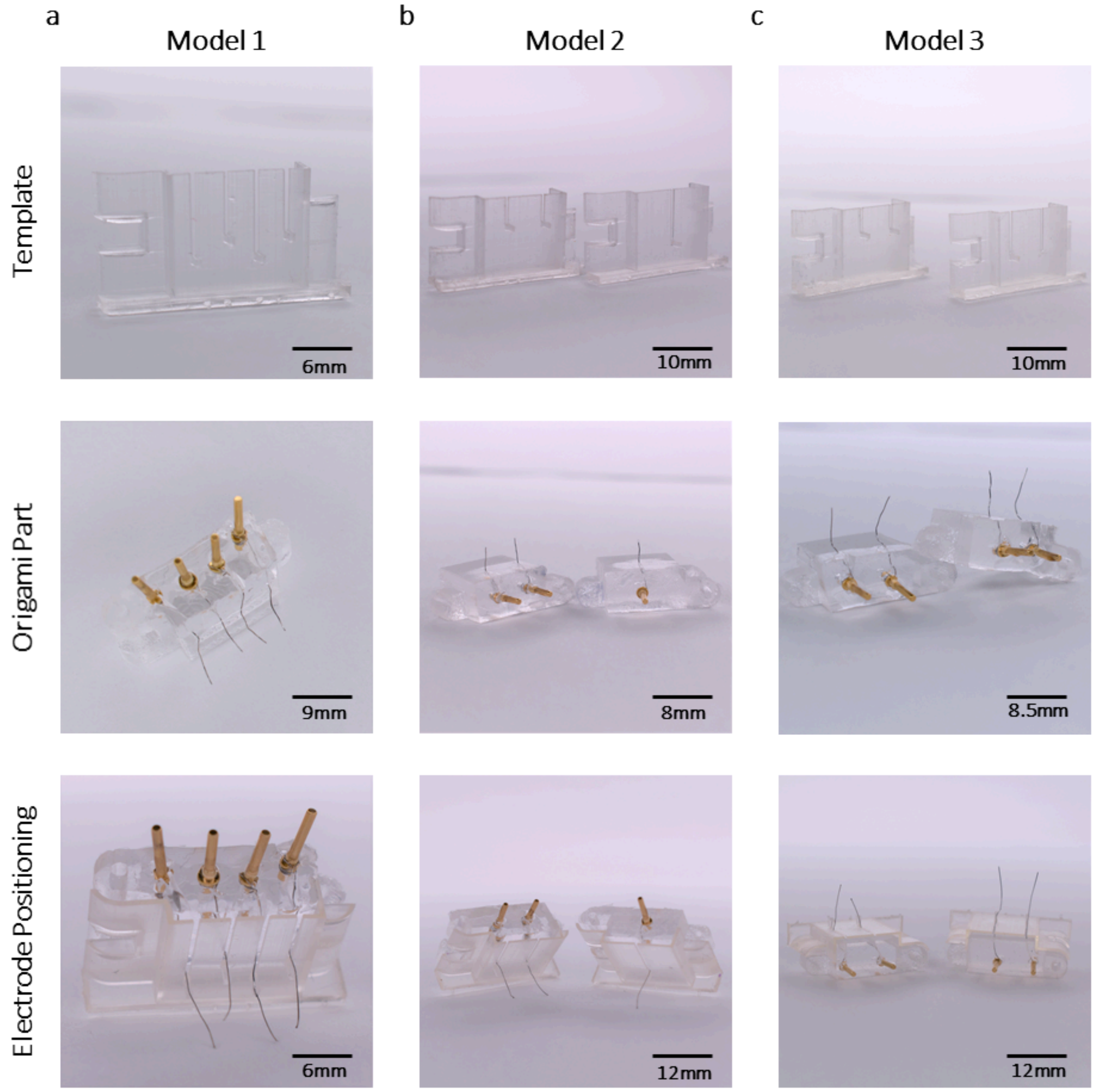

Figure S3. Electrode positioning with a custom-made template for models **a), b), and c).**

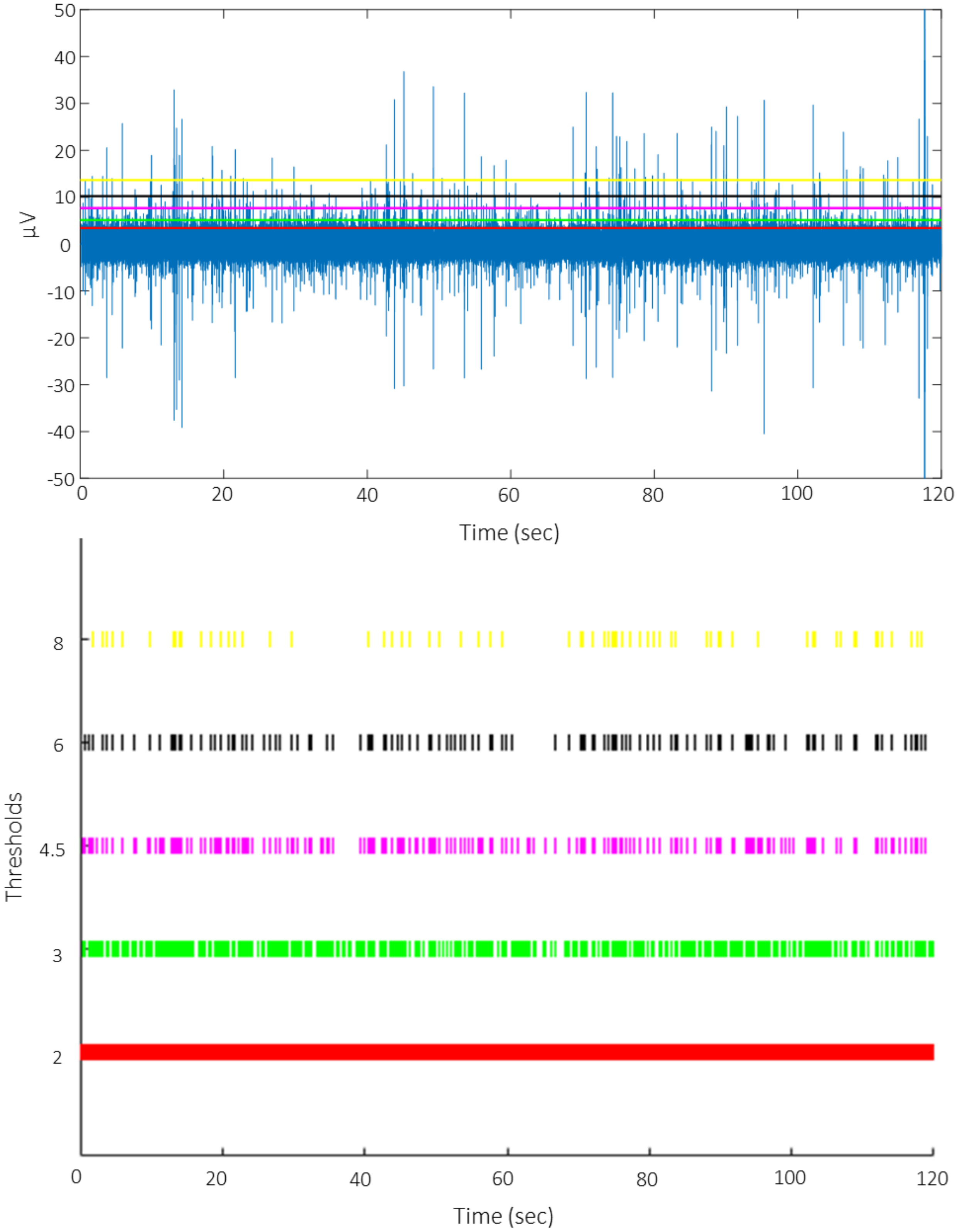

Figure S4. Spike Detection Thresholds

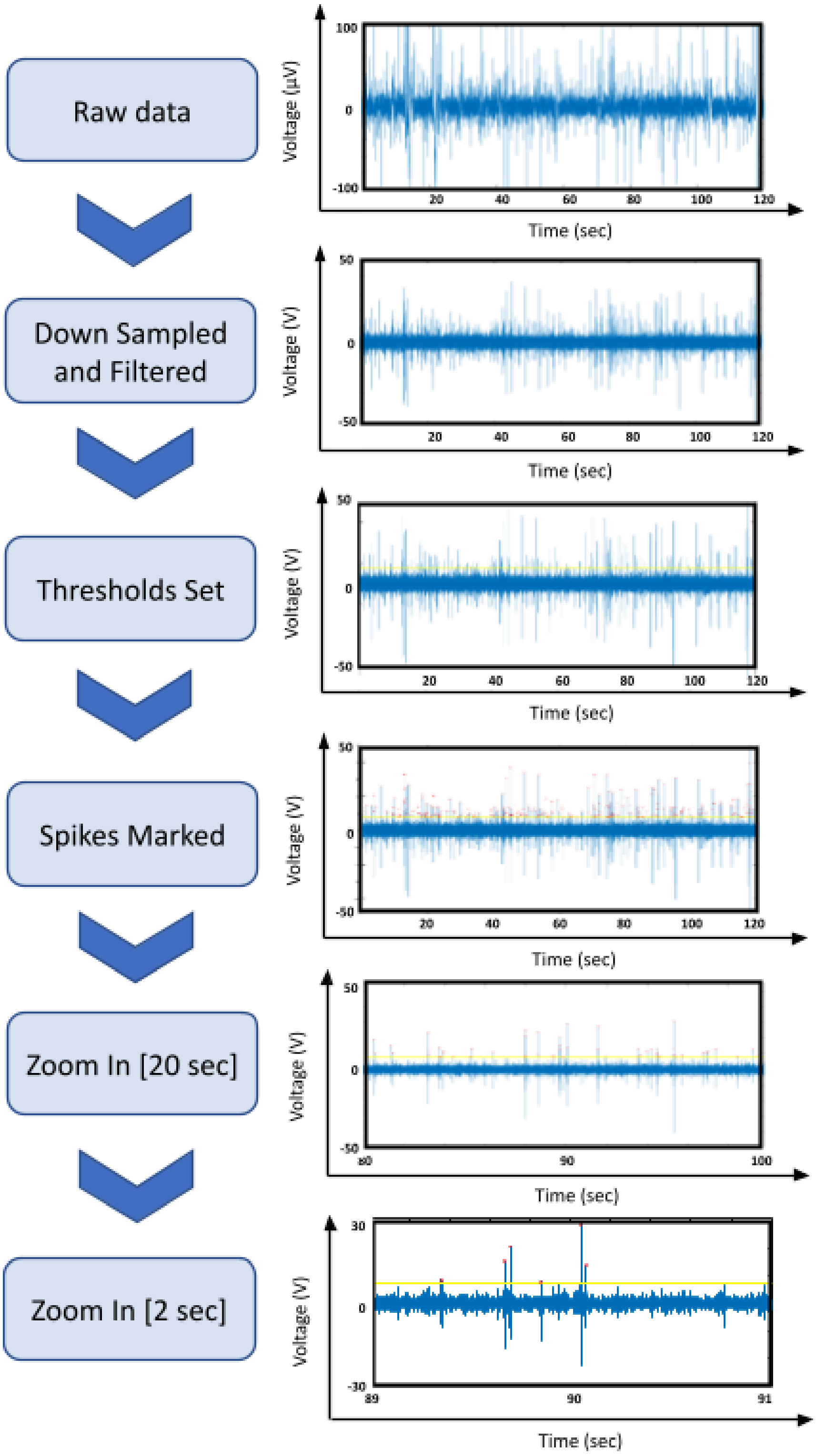

Figure S5. Electrophysiological analysis process.

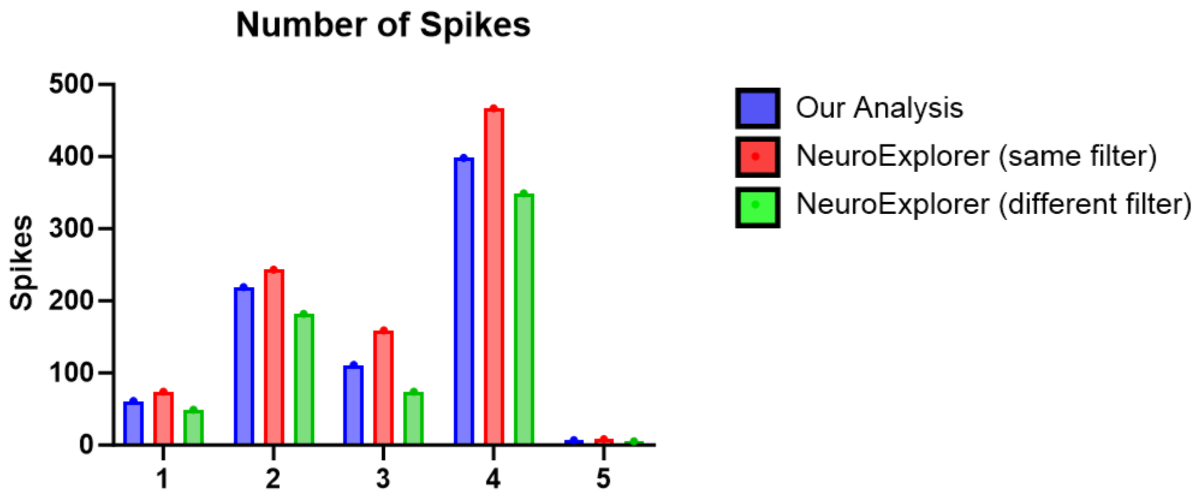

Figure S6. Comparison to NeuroExplorer - Neurons

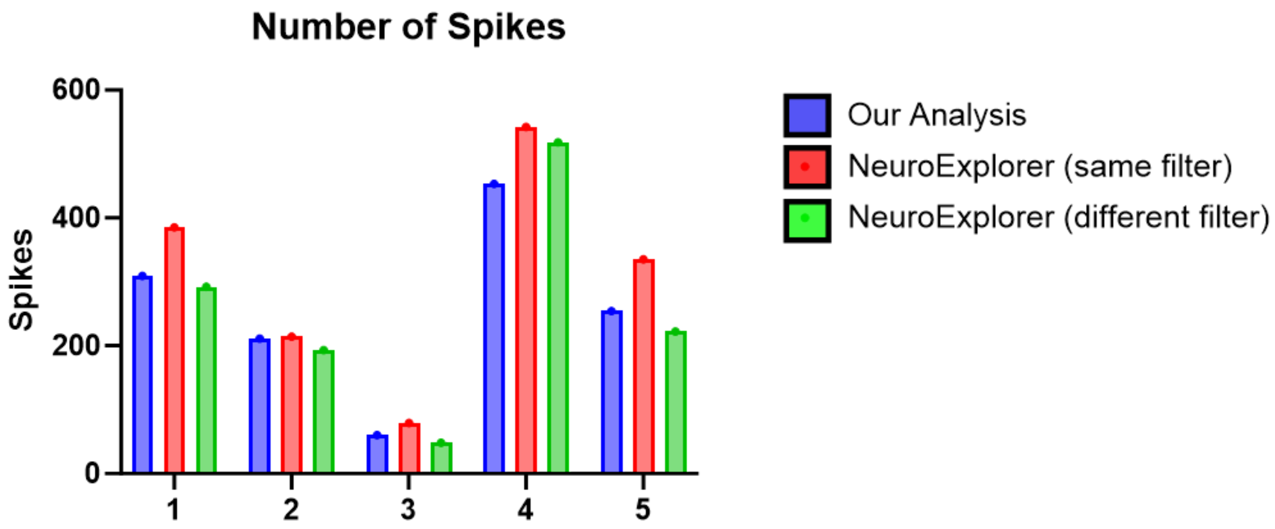

Figure S7. Comparison to NeuroExplorer – Neuron Co-Culture

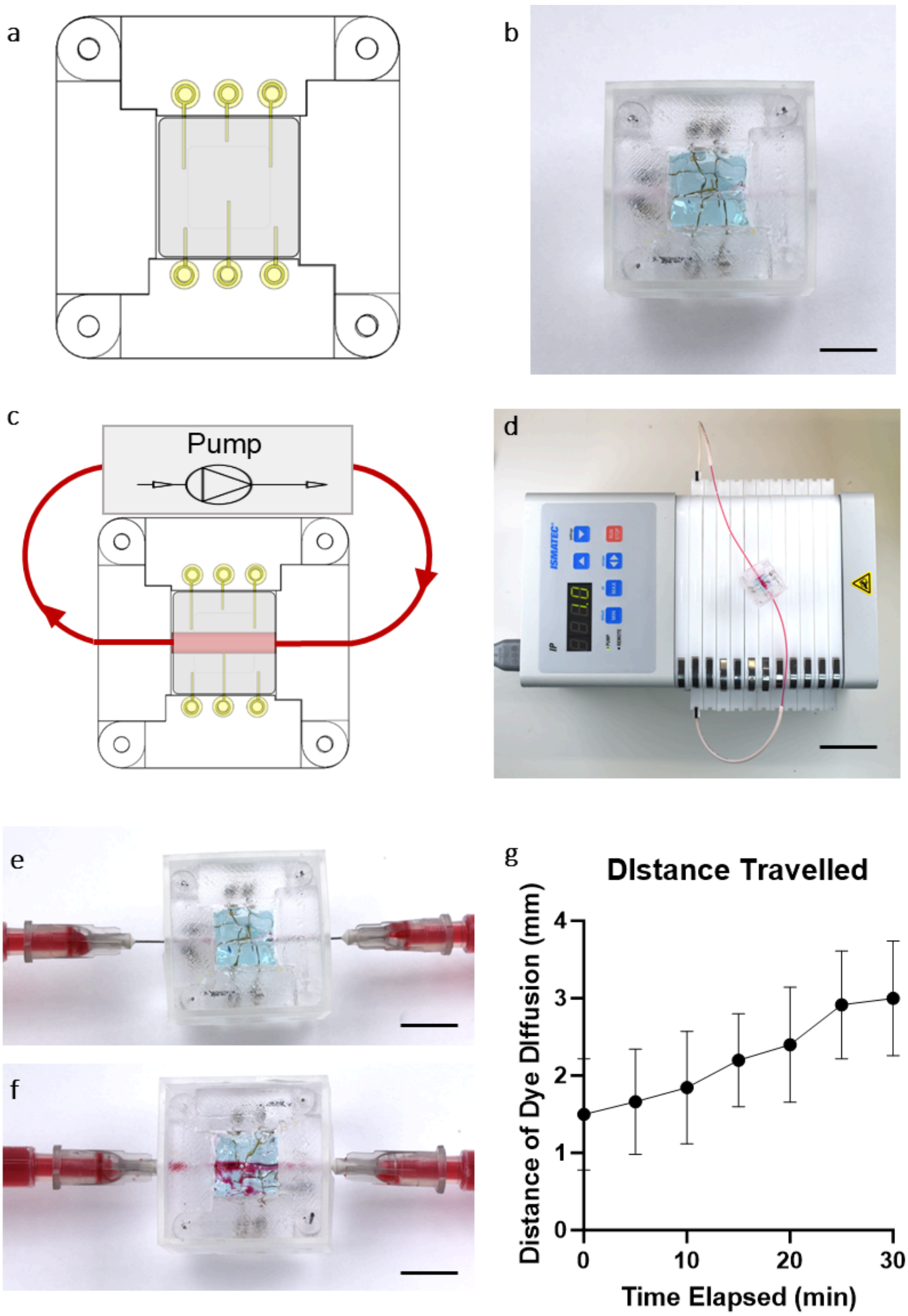

Figure S8. MSOP system under perfusion. a) Schematic representation of the origami system. **b)** Real image of the MSOP system (scale bar = 6.5mm). **c)** Schematic representation of the MSOP system under perfusion. **d)** Real image of the MSOP system under perfusion with a peristaltic pump (scale bar = 4mm). **e)** MSOP system with the inlet connected (scale bar = 7.5mm). **f)** MSOP system under flow (scale bar = 7.5mm). **g)** Measurement of dye diffusion distance over time.

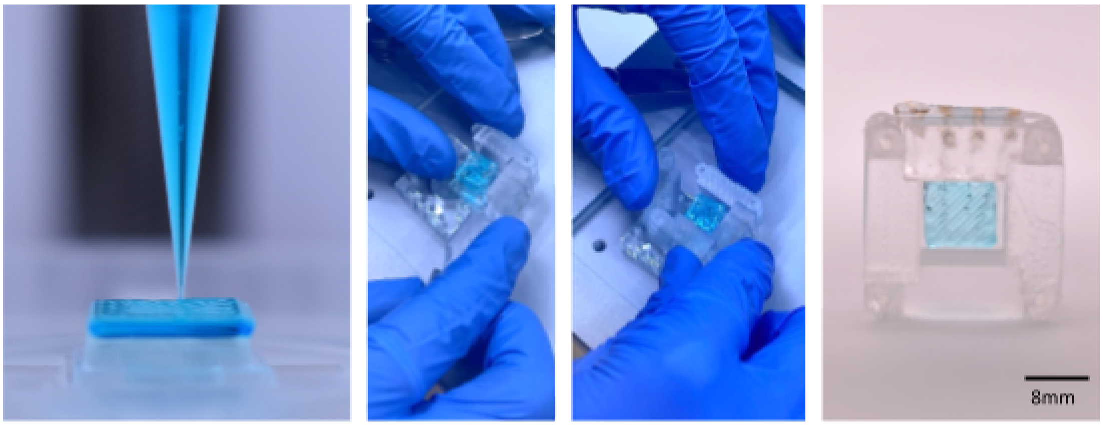

Figure S9. Custom 3D sensing device assembly pipeline with 3D bioprinted structure.

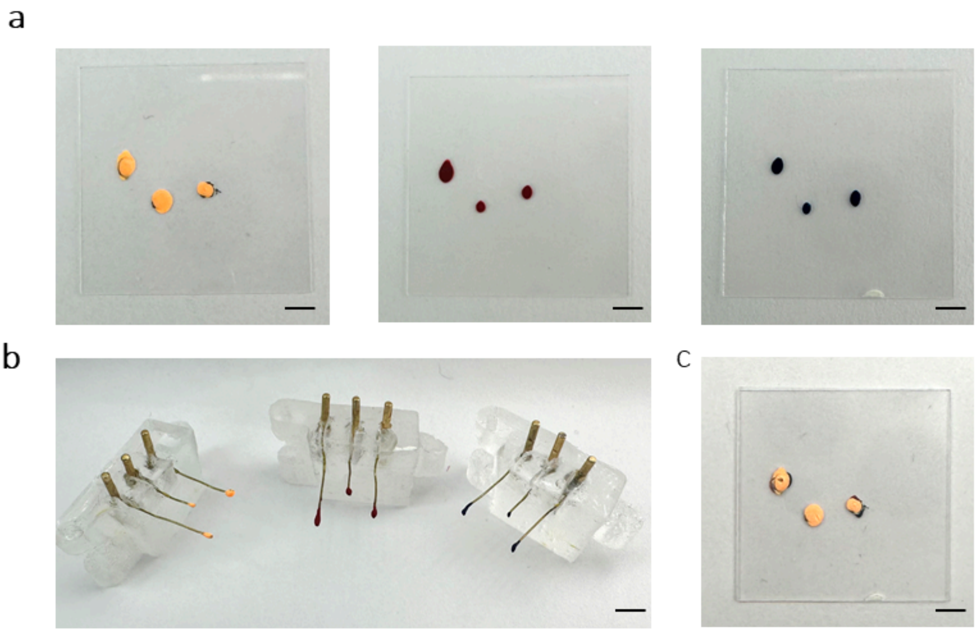

Figure S10. Electrodes accuracy test. **a)** Pictures of three stamps from three origami walls after positioning the electrode with the same holder (scale bar = 2.5mm). **b)** Figure of three origami walls after positioning the electrode with the same holder and staining the electrodes with different colors (scale bar = 4mm). **c)** Overlay of all three stamps, one on top of the other (scale bar = 2.5mm).

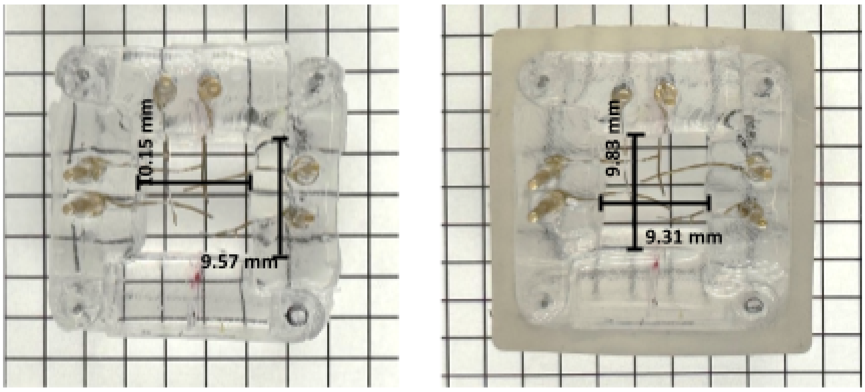

Figure S11. PDMS frame inner sizes before and after placing the sleeve.

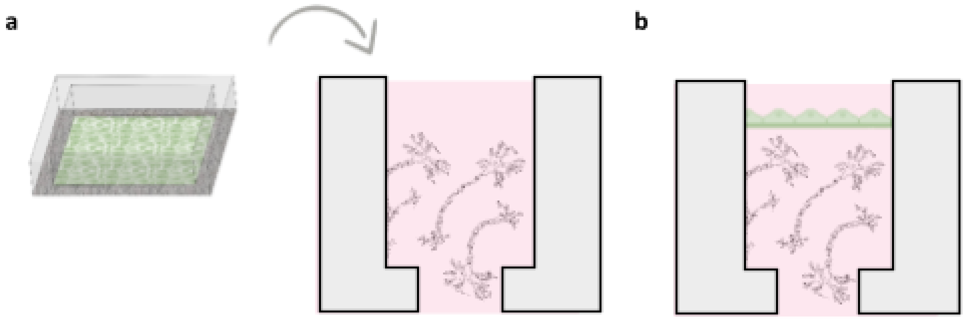

Figure S12. Illustration of the neurons and endothelial culture **a)** separately and **b)** integrated on the MSOP platform

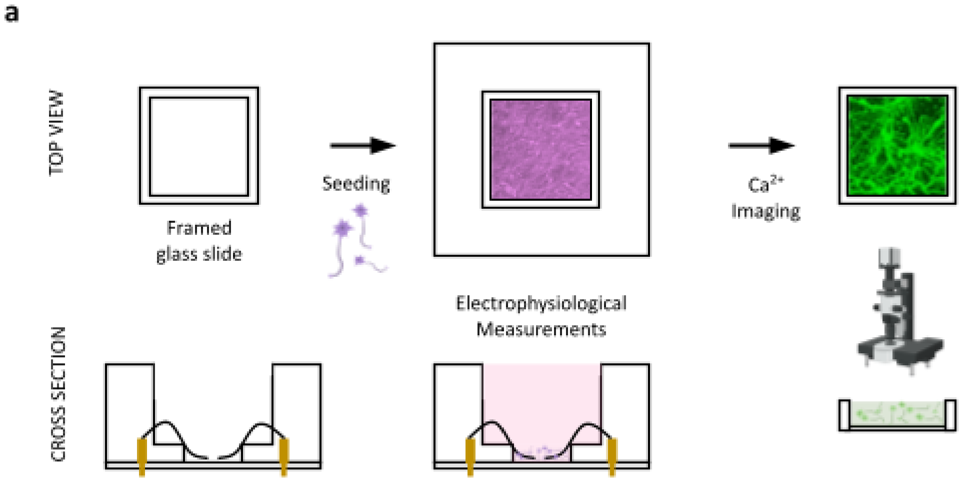

Figure S13. Top view and cross section illustration of calcium imaging on 3D neurons

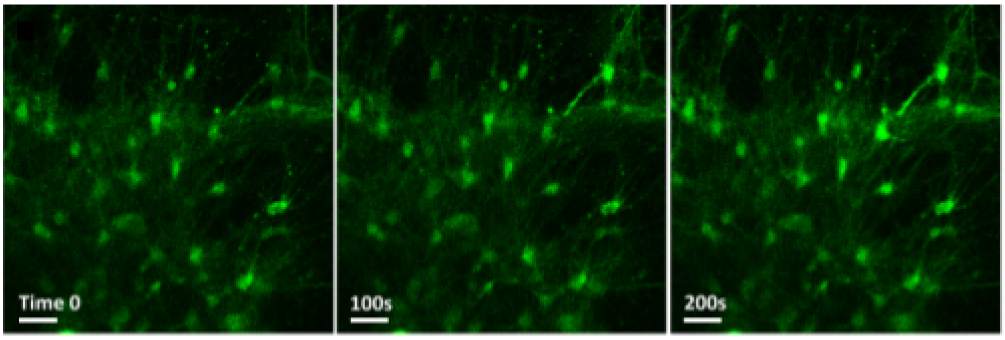

Figure S14. Images of the 3D neurons calcium imaging assay taken with a confocal microscope at time 0, 100 and 200s (scale bar = 20µm).

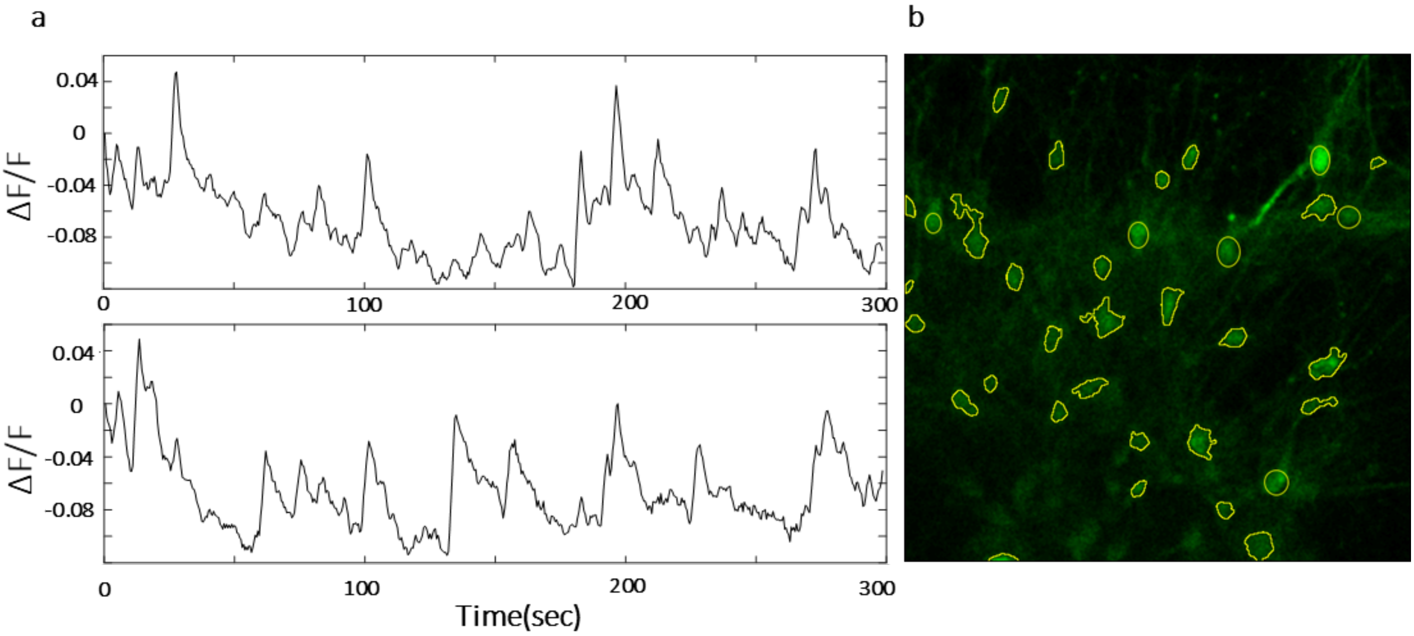

Figure S15. Calcium imaging. a) Calcium imaging traces for single cells. **b)** ROI mark of the selected cells

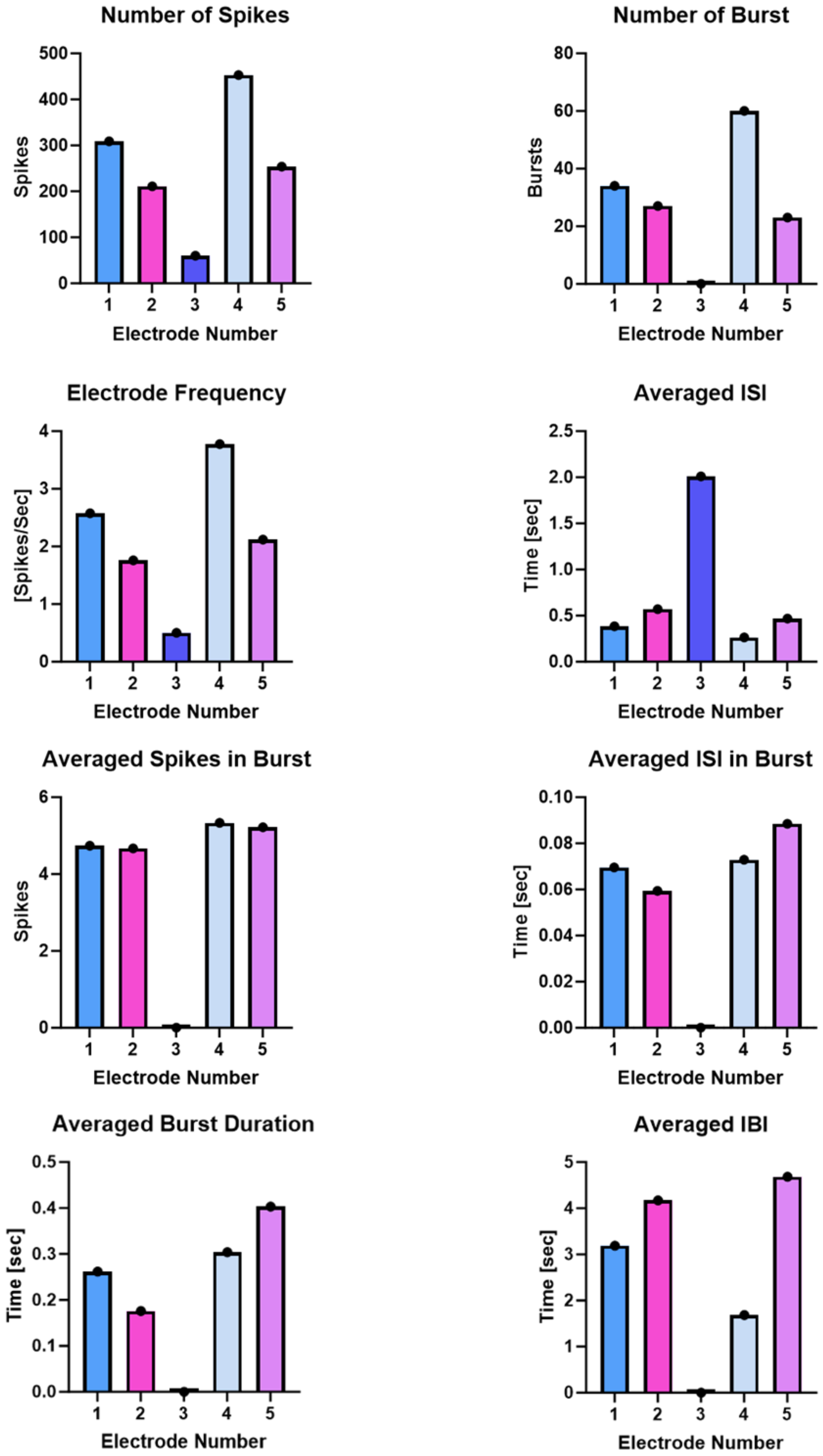

Figure S16. Electrophysiological analysis of neurons recorded with the 3D MEA after 17 days of culture in vitro.

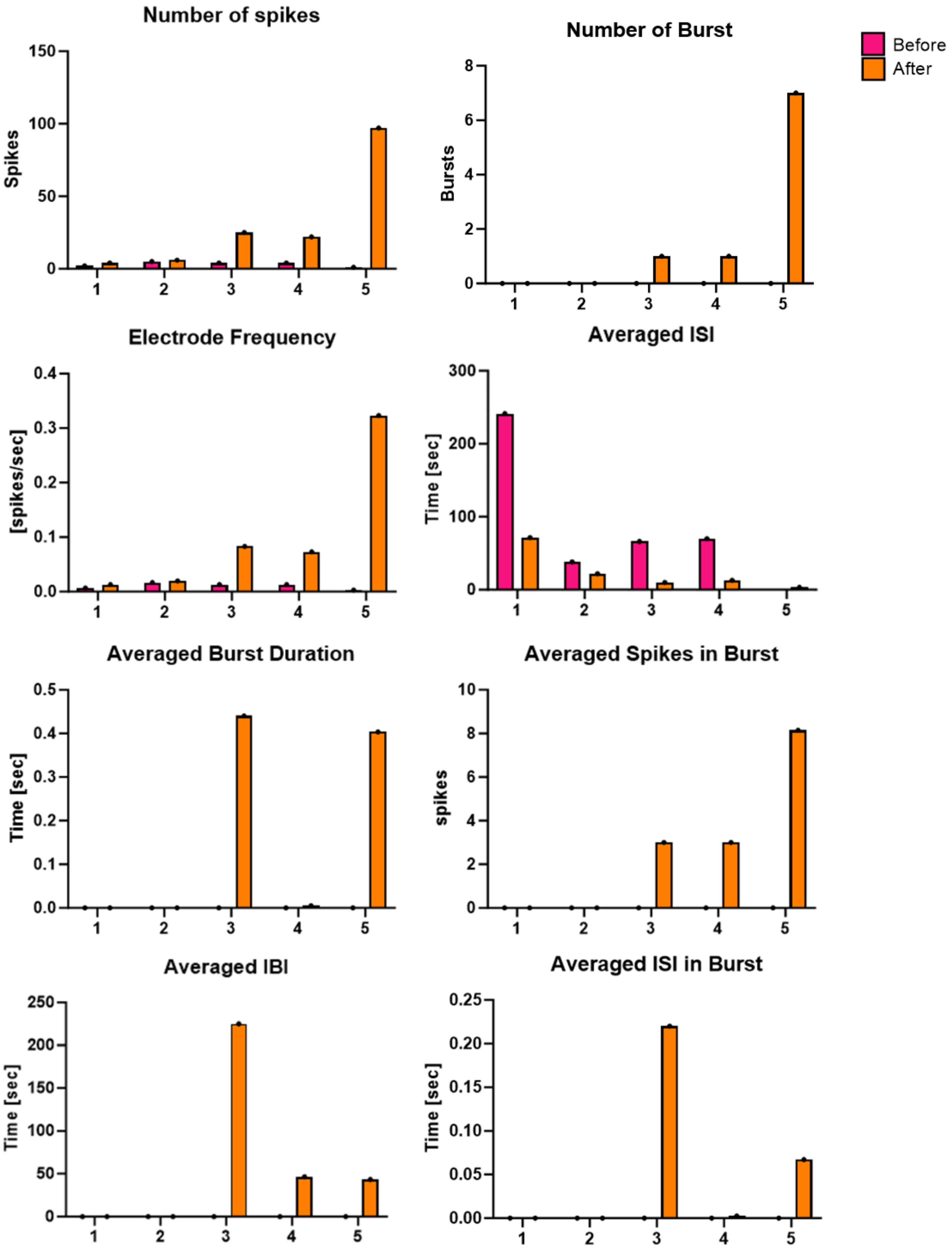

Figure S17. Electrophysiological analysis of organoids recorded with the 3D MEA before and after exposure to Bicuculline

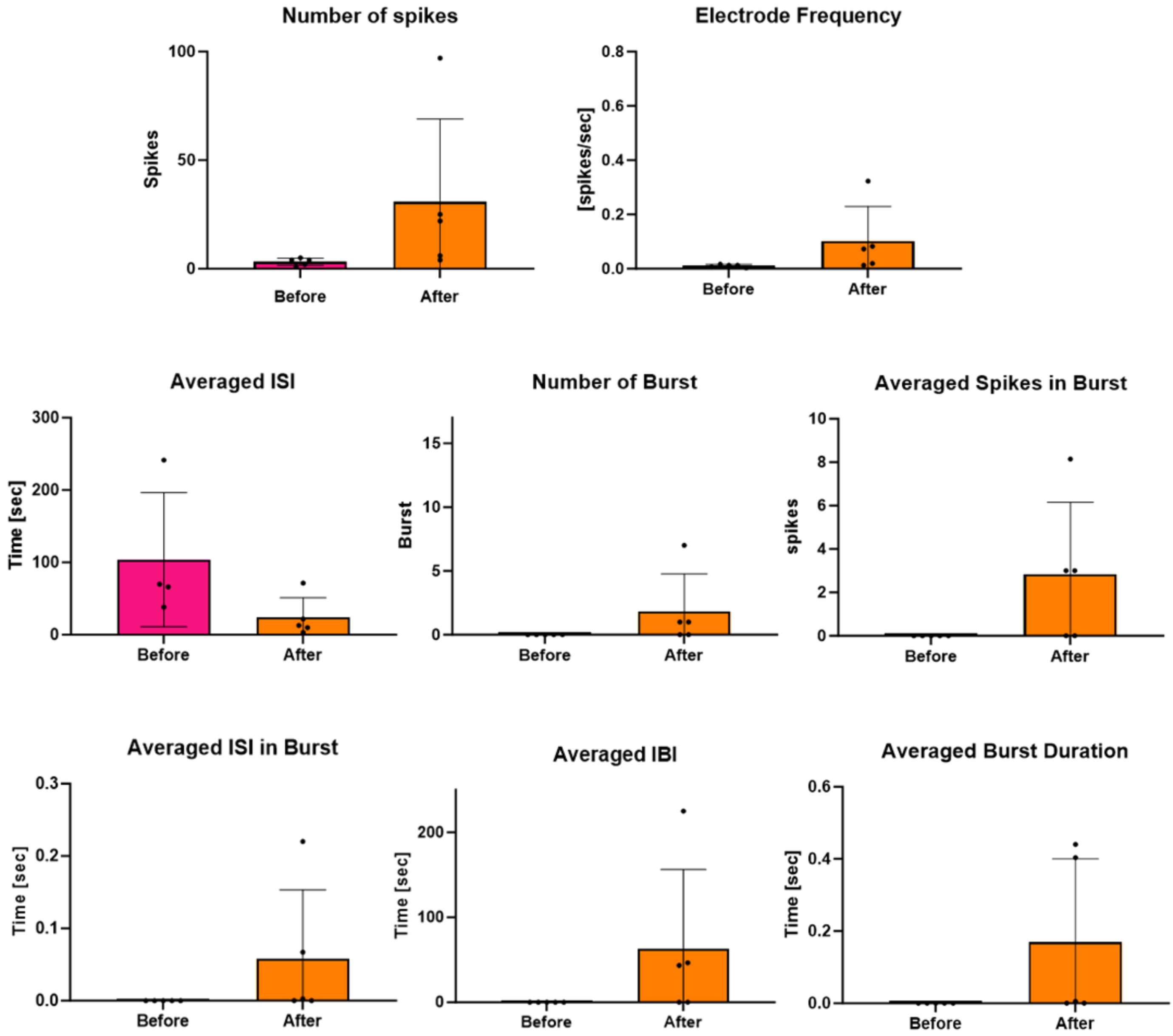

Figure S18. Electrophysiological analysis of organoids recorded with the 3D MEA before and after exposure to Bicuculline

Movies:

Movie S1. Step-by-step assembly process of the multi-sensor origami platform.

Movie S2. The organoid culture in the multi-sensor origami platform.

Movie S3. Rotation of the 3D organoid after removal of 3D electrodes.

Movie S4. The system with a 3D bioprinted model under flow.

Movie S5. Diffusion through the gel after 30 min of perfusion.

Movie S6. Calcium imaging of the 3D neurons observed with a confocal microscope.

Tables:

Reagents	Cat. No.	Manufacturer	Concentration
Hanks Modified ($Ca^{+2}Mg^{+2}$ free)	H2387-10X1L	Sigma-Aldrich	9.5g/L
$NaHCO_3$	S8761	Sigma-Aldrich	4.2mM
HEPES	03-025-1B	Biological Industries Israel Beit-Haemek, Israel	12mM
D-Glucose	G8769-100ML	Sigma-Aldrich	33mM
Kynurenic Acid	K3375-1G	Sigma-Aldrich	200uM
D-AP5(D(-)-2-amino-5-phosphop entanoic acid) (APV)	0106	Tocris	25uM
Bovine Serum Albumin (BSA)	A4919-5G	Sigma-Aldrich	300mg/L
Gentamicin	R01510	Gibco- rhenium	5ug/mL
MgSO4	9004876	Merck	12mM

Table S1- Dissection medium components (pH 7.3)

Reagents	Cat. No.	Manufacturer	Concentration
NaCl	S7653,	Sigma-Aldrich	136mM
KCl	231-211-8	Sigma-Aldrich	5mM
Na2HPO4	S5011	Sigma-Aldrich	7mM
HEPES	03-025-1B	Biological Industries Israel Beit-Haemek, Israel	25mM
NaHCO3	S8761	Sigma-Aldrich	4.2mM
Kynurenic Acid	K3375-1G	Sigma-Aldrich	200uM
APV	0106	Tocris	25uM

Table S2- Digestion medium components (pH 7.4)

Reagents	Cat. No.	Manufacturer	Concentration
Neurobasal medium	21103-049	Rhenium/Thermo Fisher Scientific	91.5%
B27	A1895601	Rhenium/Thermo Fisher Scientific	2%
Glutamax	35050038	Gibco	1%
Penicillin Streptomycin	03-031-1B	Biological Industries Israel Beit-Haemek	0.5%
FBS	F7524	Sigma-Aldrich	5%

Table S3- Plating medium components

Reagents	Cat. No.	Manufacturer	Concentration
Neurobasal medium	21103-049	Rhenium	96.5%
B27	A1895601	Rhenium	2%
Glutamax	35050038	Gibco	1%
Penicillin Streptomycin	03-031-1B	Biological Industries Israel Beit-Haemek	0.5%
Gentamicin	R01510	Gibco- rhenium	1:500
Fudr	AC227601000	Thermo Scientific	1:1000

Table S4- Neuron growth medium components

Supporting files:

File S1. MaozAnalyzer App – MATLAB code that receives the electrophysiological recording and analyzes the data.
